# Gene-Specific Substitution Profiles Describe the Types and Frequencies of Amino Acid Changes during Antibody Somatic Hypermutation

**DOI:** 10.3389/fimmu.2017.00537

**Published:** 2017-05-10

**Authors:** Zizhang Sheng, Chaim A. Schramm, Rui Kong, NISC Comparative Sequencing Program, Betty Benjamin, James C. Mullikin, John R. Mascola, Peter D. Kwong, Lawrence Shapiro

**Affiliations:** ^1^Department of Biochemistry and Molecular Biophysics, Columbia University, New York, NY, United States; ^2^Department of Systems Biology, Columbia University, New York, NY, United States; ^3^Vaccine Research Center, National Institute of Allergy and Infectious Diseases, National Institutes of Health, Bethesda, MD, United States; ^4^NIH Intramural Sequencing Center, National Human Genome Research Institute, National Institutes of Health, Bethesda, MD, United States

**Keywords:** antibodyomics, B cell ontogeny, broadly neutralizing antibody, mutation frequency, repertoire diversity

## Abstract

Somatic hypermutation (SHM) plays a critical role in the maturation of antibodies, optimizing recognition initiated by recombination of V(D)J genes. Previous studies have shown that the propensity to mutate is modulated by the context of surrounding nucleotides and that SHM machinery generates biased substitutions. To investigate the intrinsic mutation frequency and substitution bias of SHMs at the amino acid level, we analyzed functional human antibody repertoires and developed mGSSP (method for gene-specific substitution profile), a method to construct amino acid substitution profiles from next-generation sequencing-determined B cell transcripts. We demonstrated that these gene-specific substitution profiles (GSSPs) are unique to each V gene and highly consistent between donors. We also showed that the GSSPs constructed from functional antibody repertoires are highly similar to those constructed from antibody sequences amplified from non-productively rearranged passenger alleles, which do not undergo functional selection. This suggests the types and frequencies, or mutational space, of a majority of amino acid changes sampled by the SHM machinery to be well captured by GSSPs. We further observed the rates of mutational exchange between some amino acids to be both asymmetric and context dependent and to correlate weakly with their biochemical properties. GSSPs provide an improved, position-dependent alternative to standard substitution matrices, and can be utilized to developing software for accurately modeling the SHM process. GSSPs can also be used for predicting the amino acid mutational space available for antigen-driven selection and for understanding factors modulating the maturation pathways of antibody lineages in a gene-specific context. The mGSSP method can be used to build, compare, and plot GSSPs^1^; we report the GSSPs constructed for 69 common human V genes (DOI: 10.6084/m9.figshare.3511083) and provide high-resolution logo plots for each (DOI: 10.6084/m9.figshare.3511085).

## Introduction

The variable regions of B cell receptors are responsible for antigen recognition and are generated by V(D)J recombination in the bone marrow. This generates a diverse initial pool of B cell receptors, but their affinities for antigens are usually low. Thus, somatic hypermutation (SHM) in the immunoglobulin (Ig) variable region is a key process for increasing affinity. SHM mainly occurs during B cell proliferation at the germinal center and is predominantly initiated by activation-induced cytidine deaminase (AID) (1–5), which deaminates cytosine to uracil. The resulting U•G mismatch undergoes error-prone or error-free repair by DNA repair pathways or by DNA replication (3, 6–9), and a mutation can be introduced at the targeted U•G pair or a downstream A•T pair (6, 10). AID preferentially acts at hotspot motifs such as WRCY (W = A or T, R = A or G, Y = C or T) (5, 11, 12), while avoiding coldspot motifs such as SYC (S = C or G) (13). Thus, the distribution of hotspot and coldspot motifs, or the intrinsic mutability of a germline gene, modulates where mutations occur. Moreover, the targeted nucleotide positions are more likely to result in transition mutations (A↔G, C↔T) than transversion mutations (A↔C, A↔T, C↔G, G↔T) (8), further indicating that the types and frequencies of mutations (mutational space) are not sampled randomly at the nucleotide level.

Several previous studies have attempted to capture these biases with models based on 2-, 3-, 4-, 5-, and 7-nucleotide motifs (14–19) or based on observed amino acid substitutions (20). Such substitution models provide important means for annotating antibody sequences (21), calculating genetic diversity (20) and evaluating selection pressure (22, 23). Nonetheless, recent studies have revealed that the mutability of a nucleotide motif can vary between complementarity determining regions (CDRs) and framework regions (FWRs) (12) and that mutability and mutation biases are position, chain, and species dependent (17, 24). Thus, models considering all context-dependent factors are required to characterize the SHM process precisely.

Patterns of amino acid mutations resulting from SHM biases at the nucleotide level are of great interest, as they determine antibody functionality. However, the subset of possible amino acid changes that are explored during affinity maturation has not been systematically investigated. This is partially because variations in the mutability and substitution bias of the three nucleotide positions of a codon lead to complications in the prediction of mutability and substitution biases of an amino acid position. In addition, the effects of functional selection are difficult to predict *a priori*, especially when the cognate antigen is not already known. Nonetheless, in a previous study, we observed that antibodies originating from the same germline V gene shared ~20% of their amino acid substitutions in the V region, irrespective of antigen specificity (25). Similarly, in a recent vaccination study, we showed that the high-frequency substitutions observed in the V region of IGHV1-2-derived anti-Env antibodies also appear with high frequency in IGHV1-2-derived antibodies that do not target Env (26). Together, these results suggest that the occurrence frequencies of amino acid substitutions in antibody repertoires are at least partially independent of antigen-driven selection, and that the intrinsic mutability and substitution bias of a germline gene is a dominant factor modulating SHM. This suggests the possibility of using gene-specific substitution profiles (GSSPs), which implicitly incorporate all context-dependent factors regulating SHM, to predict the mutational space sampled by SHM machinery. Such investigation is important for understanding substitution patterns observed in antigen-specific antibodies and predicting the chance of re-eliciting similar SHM patterns by vaccination.

In this study, we describe a new software tool for examining the sampled amino acid mutational space for each position of germline V genes. We demonstrate the usefulness of this technique by analyzing the antibody repertoires of six human donors and demonstrating that the GSSPs constructed using substitutions from functional antibodies and recapitulate the mutational space sampled by SHM machinery. We show that the mutational space of a V gene is not sampled uniformly and that the sampling bias is gene specific and similar among donors and over time. The software for constructing and analyzing GSSPs is available from GitHub as part of the SONAR suite (27).

## Results

### Construction of Robust GSSPs for V Genes

**Figure 1:**
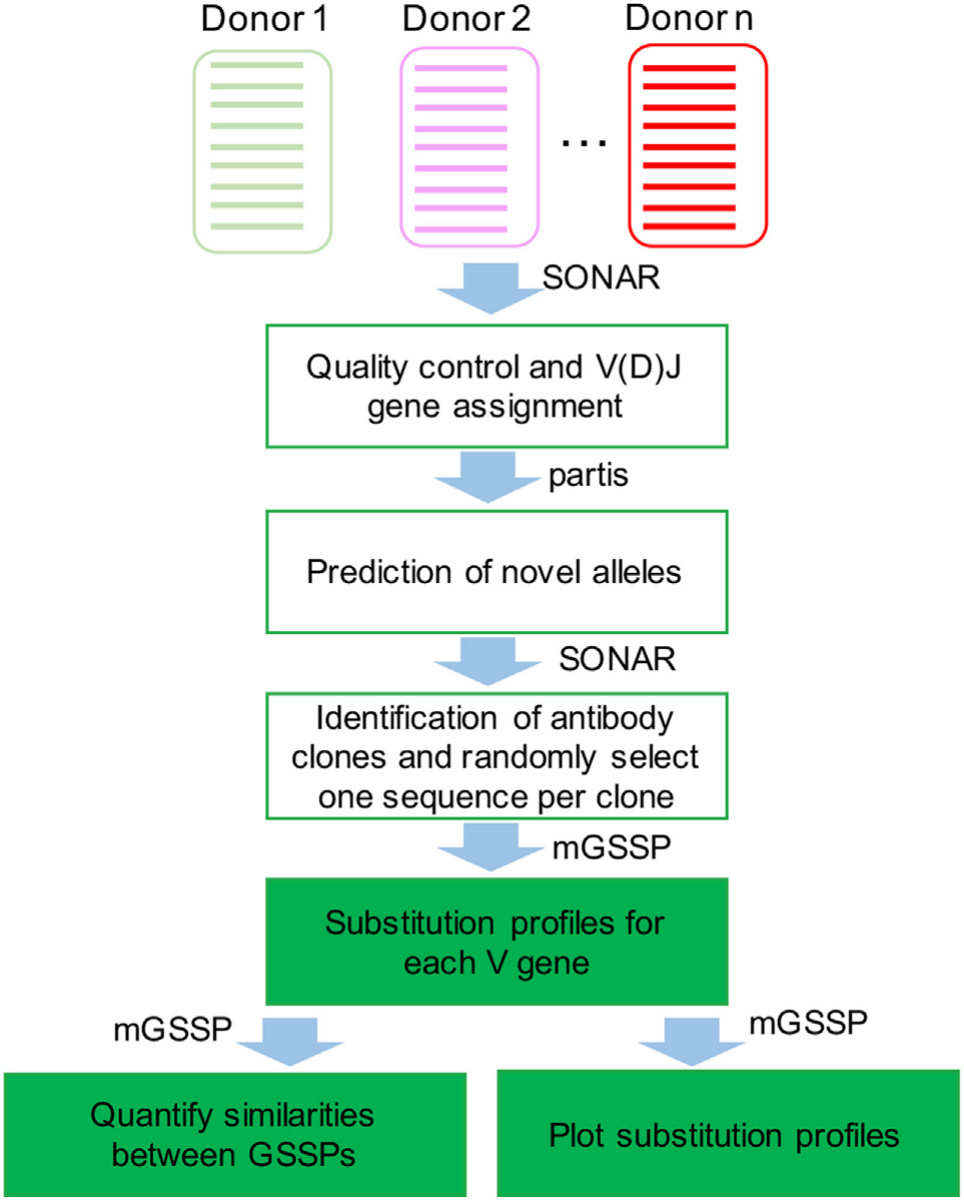
**Flowchart for the analyses of human antibody repertoires and construction of gene-specific substitution profile (GSSP)**. The next-generation sequencing datasets were first processed using SONAR to filter out low-quality reads and to assign V(D)J genes for each transcript. Novel alleles were identified using partis. SONAR was then used to identify antibody clones, and one representative sequence was chosen in each clone for building GSSPs using method for gene-specific substitution profile.

**Figure 2:**
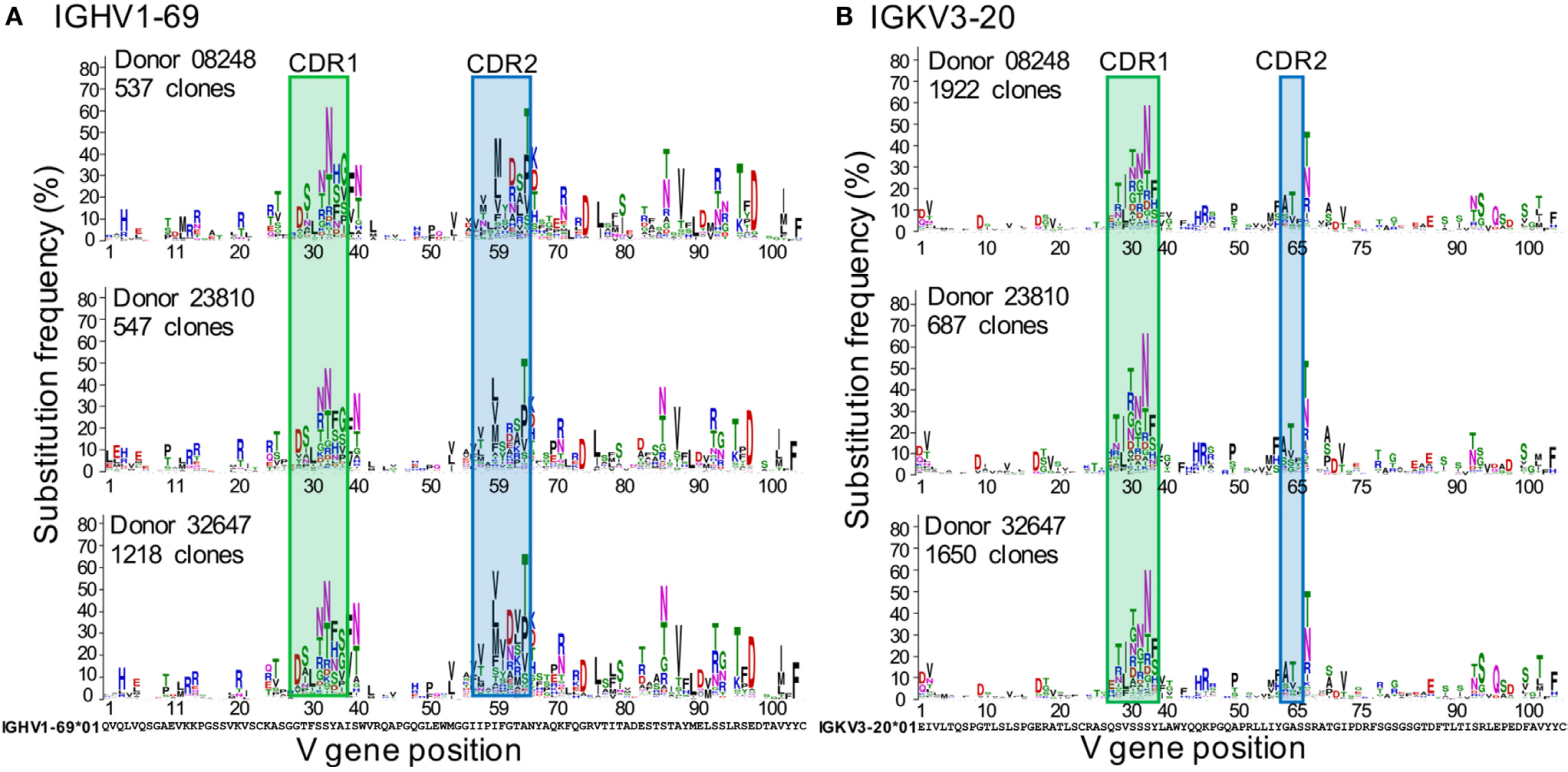
**Inter-donor similarities of gene-specific substitution profiles (GSSPs) of IGHV1-69 and IGKV3-20 germline genes**. At each position of the GSSPs, the length of an amino acid letter represents the frequency of substitution observed in a repertoire, with the germline amino acids showed at the bottom of each panel. For each gene, the GSSPs [**(A)** for IGHV1-69 and **(B)** for IGKV3-20] are similar among three healthy donors. For each position of each donor, the substitutions were colored by the physicochemical properties of the amino acids. Blue: Arg, Lys, His; red, Asp, Glu; green, Gly, Ser, Thr, Tyr, Cys; black, Pro, Ala, Trp, Phe, Leu, Ile, Met, Val; purple, Asn, Gln. See also Figures S1 and S2 in Supplementary Material.

To investigate the mutational space sampled by each germline V gene, we first compared the somatic mutations observed in human antibody repertoires of three healthy donors and three HIV-1-infected donors. Briefly, B cell receptor transcripts from peripheral blood B cells were sequenced using either Roche 454 pyrosequencing or Illumina MiSeq technologies. Starting from hundreds of thousands or millions of reads from each sample (Table S1), we assigned germline V and J genes for each transcript, removed low-quality sequences, identified clonal lineages, and selected one representative sequence per clonal lineage to build GSSPs for each V gene (Figures 1 and 2). We used the program partis (21) to predict novel germline alleles for all quality-filtered repertoires (see Materials and Methods), providing high confidence that all germline gene polymorphisms were excluded from the profiles.

In order to test the robustness of GSSPs to noise in the data, we subsampled the lineages found in each of the three healthy donors and built profiles for common VH genes using 25, 50, 100, 200, or 300 lineages per profile. We then calculated the Jensen–Shannon divergence between GSSPs (see Materials and Methods). We found that the between-donor Jensen–Shannon divergence between these profiles began to converge when 300 lineages were used to create a profile (Figure S1A in Supplementary Material). We therefore used 300 as the minimum number of lineages to build a mutational profile for further analyses except for quantifying the similarities of GSSPs (see next section), in which seven of 69 GSSPs built using 100–300 lineages were included but they did not change our conclusions. We also determined that there were no significant differences between GSSPs from IgM and IgG repertories, and therefore treated all VH data together. High-resolution plots of each profile, as well as the underlying numerical data, can be found at DOIs 10.6084/m9.figshare.3511083 and 10.6084/m9.figshare.3511085.

For each position of each germline V gene (numbered using IMGT scheme), we calculated the rarity of all possible substitutions as

(1) $$r_{Vi,a}=1-(m_{V,i}*f_{Vi,a}\text{​})$$

where *m_V,i_* is the substitution frequency of position *i* in germline gene *V* and *f_Vi,a_* is the substitution bias, or frequency at which a particular non-germline amino acid *a* was observed in all *V* gene lineages with substitutions at position *i*. The rarity is undefined for germline amino acids and equal to 1.0 for substitutions that are never observed in a particular dataset. When we examined the effects of choosing different representative sequences for each lineage (see Materials and Methods), the rarity scores of all substitutions were highly correlated regardless of representative sequence (Figure S1B in Supplementary Material), demonstrating again that the observed GSSPs are not significantly affected by noise in the data.

### Substitution Profile of Each Germline V Gene Is Similar among Donors and Over Time

Figure 2 shows the GSSPs of IGHV1-69 and IGKV3-20 from three healthy donors in which the distributions of somatic mutation levels of lineages for each gene are similar (Figure S2A in Supplementary Material). Overall, the amino acid GSSPs of each V gene are remarkably consistent, similar to previous studies of mutations and selection (12, 18, 19, 21) In contrast, different V genes have noticeably divergent profiles, even within a single donor, as can be seen from the GSSPs of IGHV1-2 and IGHV1-69 (Figures S2B,C in Supplementary Material). The GSSPs depend on two factors, substitution frequency and substitution bias, each of which is dealt with separately in the following sections. We also combine these two to define rarity, shown in Equation 1.

The substitution frequency at each position of each V gene is similar among donors. As expected, the substitution frequency is higher within CDR 1 and CDR2 (Figure 2, as defined by IMGT), consistent with the fact that the CDRs contain higher proportions of AID hotspot motifs (18). For IGHV1-69, six positions in framework region (FWR) 3 exhibited substitution frequencies as high as positions in the CDRs (≥30%), probably because substitutions in the FWRs can play important roles in recognizing antigens and regulating antibody structural stability and conformations (28–31). Moreover, we also observed high frequencies of substitutions at positions adjacent to the CDRs (position 39 of IGHV1-69 and position 66 of IGKV3-20, IMGT numbering). Since these positions connect the CDRs to the β-strands of the FWRs, it is possible that these positions are important for regulating the flexibility and conformations of the CDR loops (28, 29).

**Figure 3:**
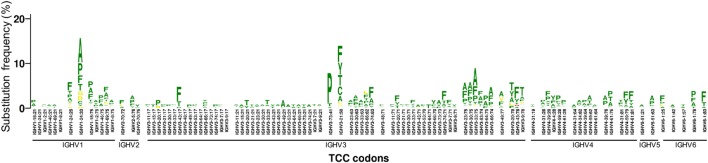
**Substitution frequency and substitution bias of codon TCC in different VH genes**. The gene-specific substitution profiles (GSSPs) of all TCC codons (encoding Serine) in our VH germline database. Codons were first sorted by VH family. Within each VH family, the GSSPs of homologous positions of different VH genes are shown together while those of non-homologous positions are separated by a space. The comparison showed that the GSSPs of TCC codons are more similar at homologous positions than between non-homologous positions, suggesting the substitution frequency and substitution bias of TCC codon are dependent on the nucleotide context. A similar nucleotide context dependency was also observed for other codons (Figures S3A,B in Supplementary Material). Color scheme: green, amino acid replacement involves single nucleotide mutation; yellow, amino acid replacement involves two nucleotide mutations; red, amino acid replacement involves three nucleotide mutations. See also Figure S3 in Supplementary Material.

For many positions, we observed 1–3 dominant substitutions with much higher frequency than other substitutions, indicating a substantial substitution bias. As expected, a major bias is toward substitutions that require only a single nucleotide change (Figure 3; Figures S3A,B in Supplementary Material). In addition, we found that amino acid substitutions requiring 2 or 3 nucleotide changes are significantly rarer than those which require only a single nucleotide change (Figure S3C in Supplementary Material). However, the exact substitutions that are preferred vary based on context, even for a specific codon (Figure 3). This is consistent with previous findings that mutation frequency varies by position within the V gene, independent of and in addition to variations due to SHM hotspots and coldspots (12, 17).

**Figure 4:**
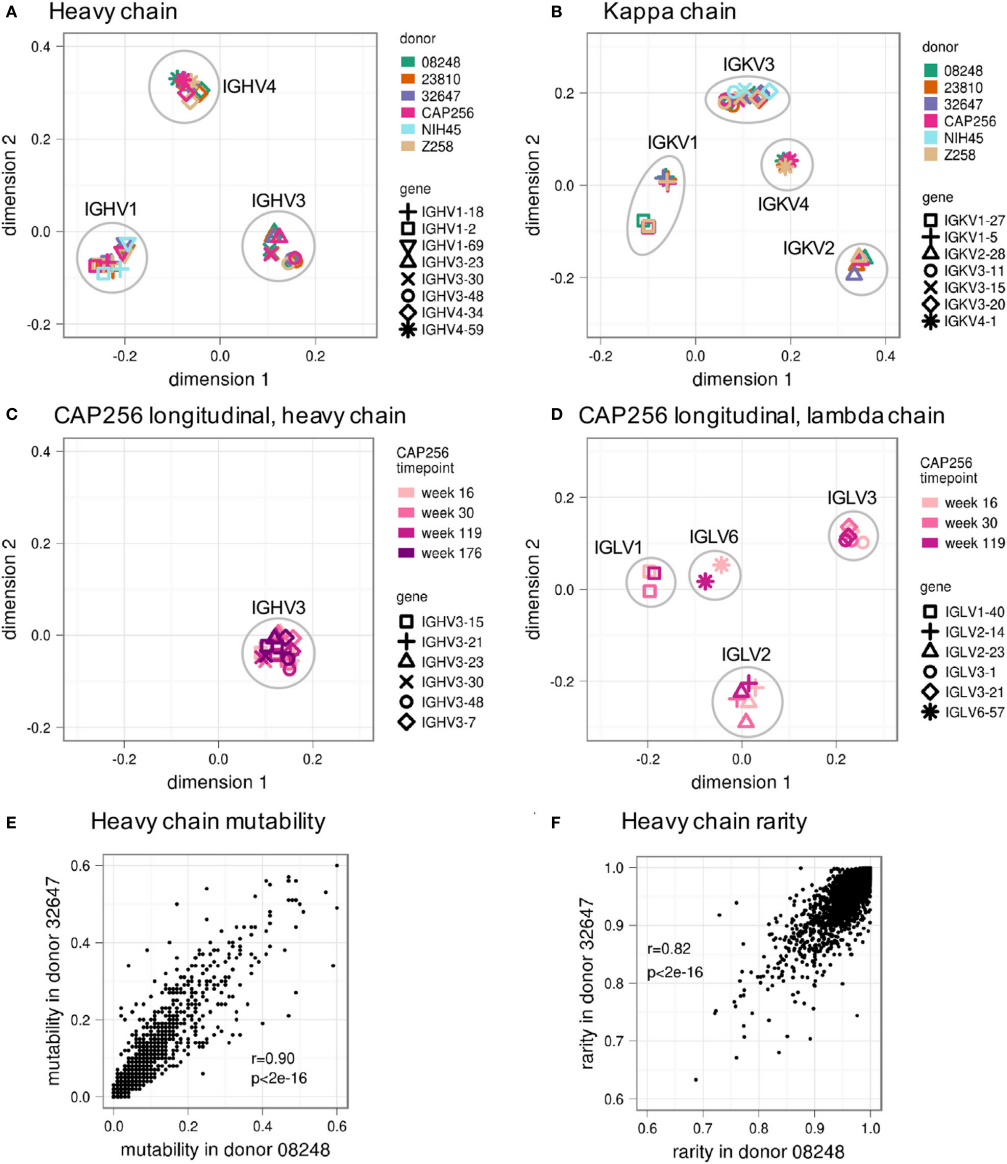
**Similarity of gene-specific substitution profiles (GSSPs) between donors and across time**. The Jensen–Shannon divergence was used to compare different GSSPs, and the resulting matrix of distances was visualized using multidimensional scaling. This showed that profiles from the same germline genes in different donors are more similar than profiles from closely related germline genes in the same donor for both **(A)** heavy chain and **(B)** kappa chain V genes. Similar analyses for lambda chain V genes are shown in Figure S4 in Supplementary Material. Note that there is no discernable difference between profiles derived from HIV^−^ donors 08248, 23810, and 32647 and those derived from HIV^+^ donors CAP256, NIH45, and Z258. Furthermore, data from donor Z258 (tan symbols) were obtained using Illumina MiSeq sequencing technology, while all other datasets were collected using Roche 454 pyrosequencing, but no difference is observed based on platform. In addition to similarity between donors, the profile from each germline gene is consistent over time, as shown for donor CAP256 **(C)** heavy chains and **(D)** lambda chains. Across all heavy chain V genes, **(E)** positional substitution frequency and **(F)** the rarity of each possible substitution are strongly correlated between donors (see also Figures S4 and S5 in Supplementary Material).

To quantitatively compare different GSSPs, we calculated weighted average of the Jensen–Shannon divergence between homologous positions over the entire V gene (see Materials and Methods). We then used multidimensional scaling (MDS) to visualize these distances for a subset of most frequently used V_H_ (Figure 4A) and V_κ_ genes (Figure 4B). (MDS plots of all V_H_, V_κ_, and V_λ_ genes are shown in Figures S4A–C in Supplementary Material). These plots confirmed that the GSSPs of each V gene from all donors clustered together and that the GSSPs of each V gene family are more similar than between V gene families. Moreover, we did not observe any differences based on HIV status or the sequencing technology used to obtain data. The GSSPs of a V gene are also similar across longitudinal samples of the same donor (Figures 4C,D; Figures S4D,E in Supplementary Material). Finally, the distributions of substitution frequency and rarity were both highly correlated between donors (Pearson’s *r* ~0.9 and ~0.8, respectively) (Figures 4E,F; Figures S5A,B in Supplementary Material). In order to increase our sampling depth, we therefore combined all lineages from the three healthy donors and generated a single set of GSSPs. We recalculated substitution frequency and rarity from these profiles, which were used for all further analyses.

### Major Factors in Determining Position-Specific Substitution Preferences Observed in GSSP

The fact that similar preferred substitutions are detected in the repertoires of multiple donors could be explained by a limited range of possibilities. One possibility, though unlikely, is that observed GSSPs are dominated by convergent selection against common antigens. It is also possible that antigen-driven selection, while critical for the development of each individual lineage, is “averaged out” over the entire repertoire such that the observed GSSPs reflect the biased action of the underlying SHM machinery. Finally, it is possible that constraints on structural stability and other sources could limit the substitutional space explored by the antibody repertoire.

**Figure 5:**
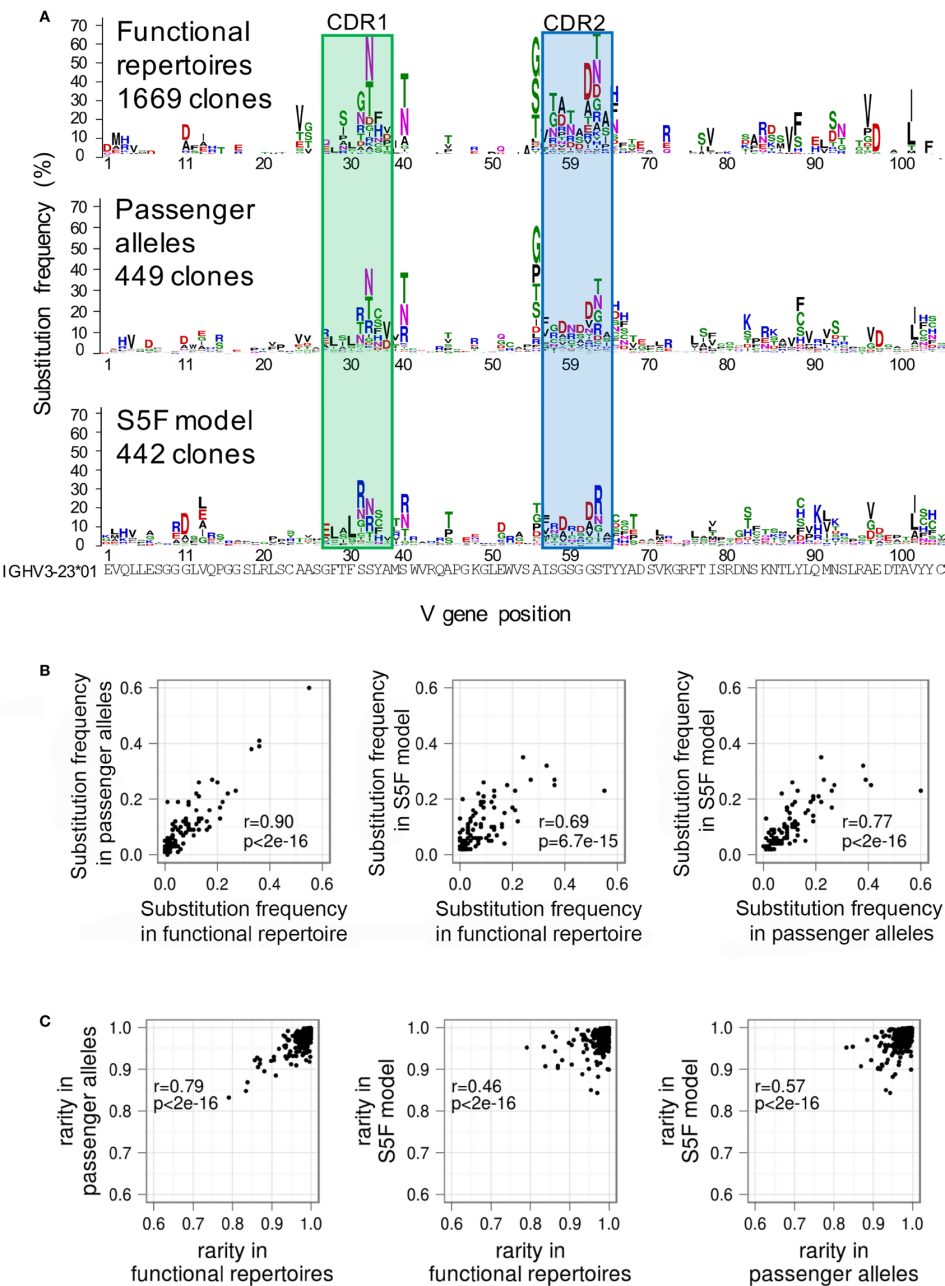
**The action of functional selection on individual lineages is averaged out when determining position-specific mutation preferences in antibody repertoires**. **(A)** Logo plots showing the IGHV3-23 gene-specific substitution profile (GSSP) for function antibody repertoires (top), non-productively rearranged passenger alleles (middle), and a simulation of SHM using the SF5 model (bottom). Despite the absence of selective pressure on the lineages derived from passenger alleles, the profile is quite similar to that of the functional repertoire. In addition, the simulated repertoire also successfully recapitulates the dominant mutations. This suggests that the action of selection on lineages in the functional repertoire is averaged out when constructing the GSSP from an entire repertoire. **(B)** Pairwise comparisons of substitution frequency among functional antibody repertoires, passenger alleles, and simulated lineages under the S5F model. The comparisons showed that the mutation frequency of an amino acid position is modulated dominantly by SHM machinery. **(C)** Pairwise comparisons of rarity among functional antibody repertoires, passenger alleles, and simulated lineages under the S5F model. The comparisons showed that the substitution bias observed at an amino acid position is modulated dominantly by SHM machinery. See also Figure S6 in Supplementary Material.

In order to distinguish between these possibilities, we first built a GSSP using lineages derived from non-productive rearrangements of IGHV3-23 (32). Because these sequences are derived from the “passenger” allele, they are not subject to selective pressure, even though AID continues to act on them (6, 33). We find that the GSSPs of the functional and non-productive repertoires are highly similar (Figure 5A), although mutations to cysteine observed at several CDR2 and FWR3 positions were suppressed in the GSSP of functional antibodies. We next compared the substitution frequency at each position of the two profiles and showed that they are as similar to each other as between functional antibody profiles of two donors (Pearson’s *r* = 0.90, *p* << 0.01) (Figures 5B and 4E). The same was true for rarity (Pearson’s *r* = 0.80, *p* << 0.01) (Figures 5C and 4F). While the residual differences between the functional and non-productive GSSPs are likely to reflect the effect of antigen-driven selection, the strong overall correlation suggests that antigen-driven selective effects are mostly averaged out when calculating GSSPs across the entire functional repertoire. Indeed, calculations of rarity based on two single lineages showed much lower correlations to their respective functional repertoires (Pearson’s *r* = 0.11, *p* = 0.11 for lineage 08248-00037 and *r* = 0.26, *p* = 1e-6 for lineage CAP256-VRC26) (Figure S6 in Supplementary Material), demonstrating that we can observe the modulation of substitution preferences for individual lineages by antigen-driven selection.

Previous work has found that, when site-specific selection coefficients for functional sequences are compared to those derived from non-productive sequences, approximately 30 and 5% of positions in the framework 3 region of human heavy chain genes are under negative and positive selection, respectively (34), which is roughly consistent between donors. McCoy et al further demonstrated that positions under negative selection tend to have less exposed surface area and proposed that the types and frequencies of substitutions at these sites may be restrained by negative selection for structural stability. To understand whether selection for structural stability modulates the similarities of substitution preferences observed in GSSPs, we compared substitution frequencies and rarity scores in the framework 3 region of IGHV3-23 from functional and non-productive repertoires at positions inferred as being under positive, negative, or neutral selection by McCoy et al (Figure S7 in Supplementary Material). The analysis revealed that the frequency of substitutions is less correlated between the functional and non-productive repertoires for positions under either positive or negative selection (Pearson’s *r* = 0.70, 0.63, 0.30 for neutral, negative, and positive selection respectively), suggesting that selection modulates the substitution frequencies observed in GSSPs. The analysis further showed that sites under positive selection showed reduced but still significant correlation between the functional and non-productive repertoires (Pearson’s *r* = 0.49 vs. *r* = 0.67 for neutral selection), suggesting selection modulates substitution bias in GSSPs. However, we were surprised to observe an increased correlation for sites under negative selection (Pearson’s *r* = 0.78), but the correlation is comparable to the measured similarity of the rarity scores for all V gene positions between the functional and non-productive repertoires (Pearson’s *r* = 0.79, Figure 5C). We note that only ~38 sites with estimates of selection pressure by McCoy et al are also present in the set of non-productive sequences used in this study, which may allow the introduction of sampling bias. Nonetheless, the sites under neutral selection showed a high correlation coefficient between the functional and non-productive repertoires, suggesting that there are other factors modulating substitution preferences.

We next sought to determine whether the substitution frequency and substitution biases observed in GSSPs are modulated by the biased action of SHM machinery. To accomplish this, we conducted simulations and generated a virtual repertoire of IGHV3-23 lineages using S5F (18), an antibody-specific mutation model that estimates a mutability and mutation preference for each nucleotide based on the four surrounding nucleotides (two on either side). The comparisons of the GSSPs showed that the simulated repertoire reproduces many dominant mutations in the functional and passenger allele repertoires, such as G10D and V89I/L (Figure 5A). Indeed, the mutability and rarity of the simulated repertoire was reasonably similar to substitution frequency and rarity of the functional and passenger allele repertoire, respectively (Figures 5B,C). Conversely, simulations using a previous antibody-specific amino acid substitution model (AB) (20) showed a heavy bias toward the use of histidine. The Pearson’s rho of the rarity scores derived from AB compared to those derived from the functional repertoires was very low (Figure S8 in Supplementary Material). This is likely due to a number of causes, including the omission of positional effects (17) and the mixture of sequences from different loci and species used to build the model.

Since the S5F model was designed to reflect intrinsic AID hotspots and coldspots (18), the strong correlation of rarity scores between simulated and actual data provides additional evidence demonstrating that the molecular machinery of SHM plays a key role in producing the observed GSSPs. Because the substitutions observed in the GSSPs recapitulate the majority of the sampled mutational space, we will use the GSSPs to understand the features of mutations available for antigen-driven selection in analyses in the following sections.

### Most Observed Mutations Are Rare but Can Be Sampled by High-SHM Lineages

**Figure 6:**
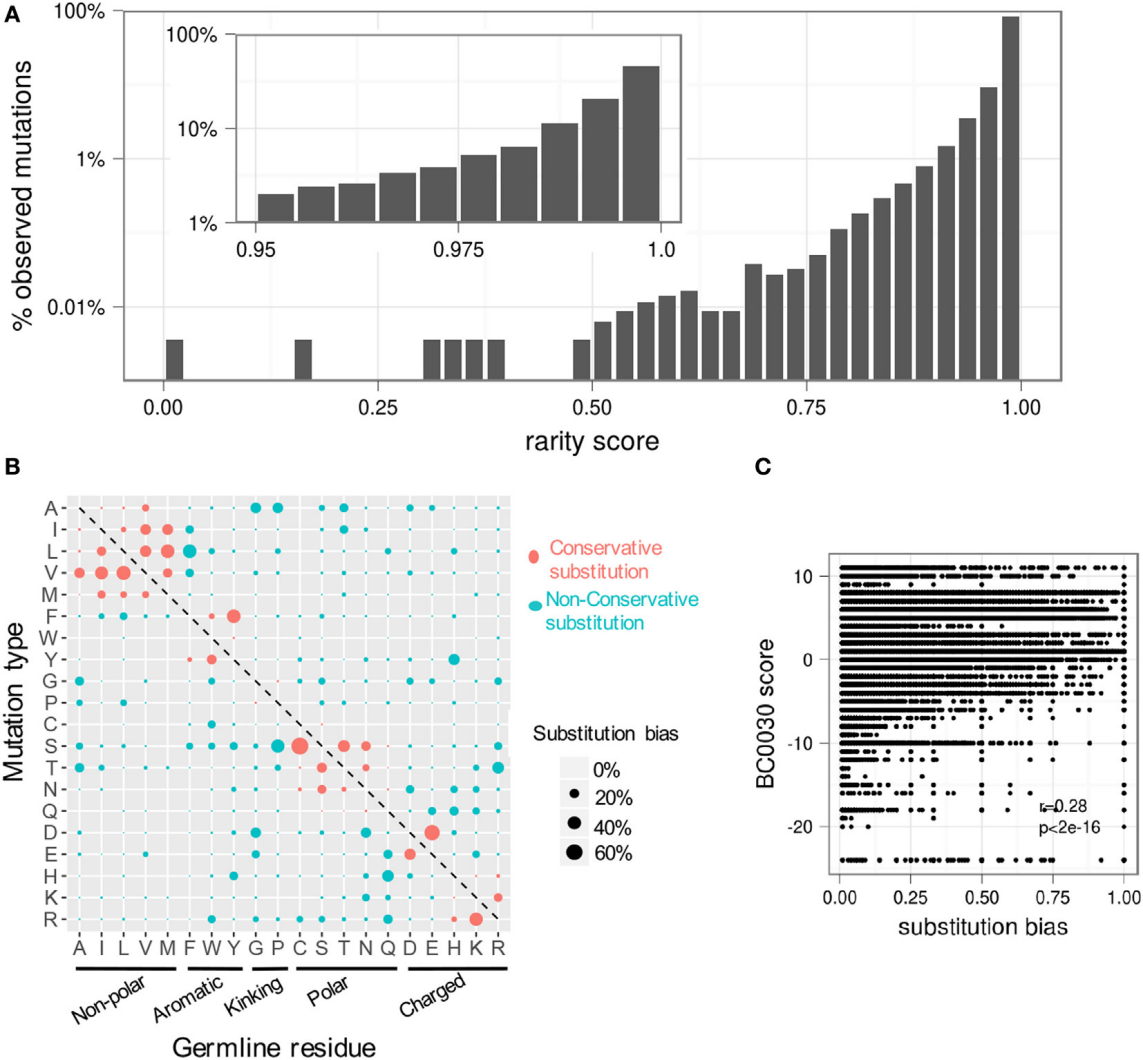
**Most somatic hypermutations in the antibody repertoire are rare**. Due to substitution biases, most observed mutations are seen only rarely. **(A)** The distribution of rarity scores for all observed mutations in all heavy chain V genes. For clarity, the top two bins are expanded in the inset. Approximately 85% of all mutations have a rarity of greater than 0.975 (rightmost bar in main plot); over 45% are extremely rare, with rarity greater than 0.995 (rightmost bar in inset). **(B)** The average substitution bias for each type of substitution shows a preference for conservative mutations and an asymmetric preference for certain mutations. **(C)** Substitution biases at individual positions are partially correlated with physicochemical properties (as measured by the BC0030 interchange matrix), but mutations with the same BC0030 score can have a wide range of substitution biases depending on genetic context. See also Figure S7 in Supplementary Material.

We next investigated the distribution of the observed rarity of all possible mutations. Strikingly, over 70% of all possible mutations were never observed in any of our datasets. Moreover, nearly 45% of observed mutations were “extremely rare” (defined as rarity > 0.995) (Figure 6A). Importantly, rarity is defined as a per-lineage score, as only one sequence per lineage is used to construct the GSSPs (Figure S1B in Supplementary Material). Thus, an extremely rare substitution is one that is expected to be sampled only by one in two 100 lineages. However, particular rare mutations can become fixed in highly expanded lineages (Figure S6 in Supplementary Material) and therefore be present in a larger fraction of individual B cells.

We then analyzed the substitution bias *f_Vi,a_* for each substitution type at positions with substitution frequency *m_V,i_* ≥ 0.05 averaged over all germline genes (Figure 6B). We used the within-position bias rather than rarity to control for differences in overall mutabilities. The results demonstrated that conservative substitutions tend to be favored, but we observed many non-conservative substitutions such as R to T and P to S. Interestingly, we also observed many substitutions that were asymmetric. For instance, R to T is more common than T to R; E to D is more common than D to E. To quantify the difference between conservative and non-conservative mutations, we compared the within-position bias for each observed substitution to its substitution score in the BC0030, a substitution matrix that scores amino acid changes based on functional interchangeability (35), which we used as a proxy for physicochemical similarity (Figure 6C). BC0030 only accounted for ~28% of the variation (i.e. Pearson’s *r* = 0.28) in within-position biases (Figure 6C), suggesting that substitutions in the antibody context are not solely constrained by physicochemical properties. In addition, the observed bias is different from a previous antibody-specific amino acid substitution model (AB model) (20), which showed high propensities for histidine to exchange with nearly all other amino acids.

While the overall distribution of SHM was similar in all three healthy donors (Figure S2A in Supplementary Material), we found that lineages with higher SHM were more likely to contain extremely rare mutations (Figure S9A in Supplementary Material). Indeed, when we subsampled IGHV1-2 lineages with either low SHM (8 or fewer amino acid substitutions; 854 lineages with a combined 3,193 substitutions from germline) or high SHM (more than 20 amino acid substitutions; 132 lineages with a combined 3,136 substitutions) from the combined dataset of the three healthy donors, we found that they resulted in substantially similar profiles (Figure S9B in Supplementary Material) and highly correlated rarity scores for substitutions that were observed in both sets of lineages (Figure S9C in Supplementary Material), after accounting for the smaller number of lineages in the high SHM set. However, the high SHM lineages contained more unique substitutions that were not observed in the low SHM set (Figure S9D in Supplementary Material). Although this may be expected, as most possible mutations are rare, it confirms that the elicitation of antibodies with specific rare mutations is expected to require higher overall levels of SHM.

## Discussion

To successfully target foreign pathogens, the immune system employs multiple mechanisms for generating diversity in the antibody repertoire, including V(D)J recombination, heavy and light chain paring, P- and N-nucleotide addition, and SHM (36). In humans, ~5 × 10^6^ new naive B cells are generated each day, and the total number of circulating B cells is ~10^11^ (36). The total number of possible distinct antibodies resulting from these processes has been estimated at up to 10^18^ (19). Hence, the antibody repertoire is so diverse that a truly random search would not allow for an effective immune response in a timely fashion. Previous studies have shown that the abovementioned processes have preferences such as biased usages of V(D)J genes for recombination and biased number of nucleotides for P- and N-additions (19, 37–39). Thus, antibodies with certain genetic signatures could occur with high probability. As a result, similar antigens can reproducibly elicit stereotypic antibodies (containing similar genetic signatures and/or somatic mutations) in many individuals [reviewed in Ref (37)]. GSSPs reveal that each V gene explores a unique subset of mutational space, which may limit the spectrum of antigens that can be recognized. Thus, the evolution of many divergent germline V genes represents an important strategy to expand the total mutational space sampled and to increase the number of antigens that can be recognized.

SHM has also long been known to operate in a biased fashion, as AID has preferences for hotspot motifs and avoids coldspot motifs (6–9, 11–13, 33). Much effort has been invested in building context-dependent models to predict the mutation propensity of Ig genes at nucleotide level (14–19, 24). These models have emphasized the difference between the generation of mutations by SHM machinery and the selection of those mutations into the functional repertoire. This distinction is critical for insight into the biological mechanisms of SHM, as well as for a proper understanding of the functional consequences of individual mutations and how affinity toward a particular antigen is increased. Nonetheless, we show that a simple model incorporating the combined effects of both processes reveals that observed substitutions have gene- and position-specific stationary frequencies.

In this study, we explored the applicability of gene-specific profiles for inferring SHM propensities of V genes at the amino acid level. We demonstrated that the sampled mutational space for each V gene is strongly constrained, giving rise to substitution frequency and substitution biases that are consistent between donors and over time. Similar consistency between donors is also observed at the nucleotide level (21). We also demonstrated that the GSSP of IGHV3-23 functional antibodies is highly correlated with that of the passenger allele profile. Thus, the GSSPs describe the mutational space sampled by SHM machinery. Because GSSPs characterize SHM propensity at each gene position, researchers lacking experience with substitution models can still use these profiles for evaluating the propensity of amino acid mutations of interests. We also have made our scripts for building, comparing, and plotting GSSPs available for researchers to build profiles from their own datasets and use GSSPs in their studies.

The analysis of GSSPs advances our understanding of the SHM process. Overall, we showed that most mutations are observed only very rarely or not at all in the repertoires we examined. While the nucleotide-based S5F model that we tested was able to capture many but not all of the dominant mutations, the GSSP of the simulated repertoire displayed differences from those calculated from actual data (Figure 5A). Moreover, since the mutability of a particular nucleotide in the S5F model can change dramatically due to changes in the surrounding bases, it is difficult to calculate the likelihood of observing a specific mature antibody sequence which could have evolved through many different paths. Conversely, GSSPs effectively sum over all possible evolutionary paths and therefore provide a basis for developing methods to directly estimate the probability of observing similar SHM patterns.

Overall, antigen-driven selection acting on individual B cells does not appear to be a dominant factor modulating the substitutions observed in the profile of the overall repertoire. This is likely because most of the effects of antigen-driven selection are canceled out when the substitutions of many lineages are incorporated. The observation that the SHM machinery generates a limited pool of available mutations with frequencies that remain mostly unchanged after selection against varying sets of antigenic challenges in different people suggests the presence of an evolutionary equilibrium between the biases of the SHM machinery and the types and positions of mutations that are most likely to produce favorable substitutions. Indeed, previous studies have shown that codon usage within antibody V genes has likely been optimized by evolution to enhance the potential for beneficial changes via SHM (40). A recent study demonstrated that a substitution F83A in light chain VK1 far away from the paratope, nevertheless appeared with high frequency in mouse antibodies, improves epitope binding affinity by regulating the torsion angle of heavy-light chain pairing (31). Thus, investigating the functional impacts of the dominant substitutions revealed by GSSPs will be important for understanding antibody affinity maturation process and antibody design. Nonetheless, differences between substitutions in functional and passenger allele repertoires can still be observed in both this study and previous work (34). McCoy et al showed that approximately 30% of positions at the framework 3 region of human heavy chain genes are under significant negative selection (34). Although varying between genes, the positions under selection are roughly consistent between donors for each gene. Because each donor experiences different infection history, this suggests that the selection may be driven by factors other than antigen specificity. One possibility is that certain mutations are systematically and consistently filtered out by selection for structural stability (34). Since GSSPs provide a pool of SHMs available for antigen-driven selection, the removal of those detrimental mutations in the mutational space will not impact the general usefulness of GSSPs, but are nonetheless of biological interest. We are currently conducting investigations of the functional effects of high-frequency substitutions which will help elucidate this relationship between SHM biases and selected substitutions.

The GSSPs presented in this study were constructed mainly using repertoire data from 454 pyrosequencing. Although the 454 pyrosequencing technique is error-prone, most errors are indels (~0.09% substitution error rate vs. ~0.9% indel error rate) (41), which cause frame shifts and can be easily filtered out (see Materials and Methods). The base substitution errors on the 454 platform are similar to or lower than substitution errors observed using the Illumina MiSeq or HiSeq platforms (~0.25% substitution error rate) (41). Moreover, we showed in Figure 4 that profiles from donors sequenced using both 454 and Illumina MiSeq (donor Z258) are highly similar. Thus, residual sequencing errors do not appear to affect our conclusions. Nonetheless, as demonstrated in Figure S1, more robust GSSPs can be constructed by incorporating more antibody lineages. Thus, we will incorporate more repertoire data to improve the prediction accuracy of the GSSPs in the future. We will also construct GSSPs for animal models including mouse, guinea pig, and non-human primates.

In this work, we have focused on substitution biases in the V genes, which comprise the bulk of the antibody variable region. However, most antibody paratopes include significant portions of the CDR3s, especially from heavy chain. Because it is difficult to assign D genes accurately and much of the CDR3 is not genetically encoded at all, the methods used in this study are not directly applicable. More accurate antibody-specific nucleotide- and amino acid substitution models that can predict the mutational space for a specific CDR3 and/or J gene region would be extremely useful and represent an important avenue for future work.

Gene-specific substitution profiles can aid interpretation of SHM patterns observed in mature antibodies and can shed light on their developmental pathways. The observation of dominant mutations suggests that the sampling of various combinations of the dominant mutations is an important and efficient mechanism to increase antigen specificity. This is consistent with our recent study showing that antibody clones elicited by the same immunogen in different donors share highly similar substitutions (26), suggesting that a dominant maturation pathway may exist. GSSPs provide a means to evaluate the probability of eliciting specific desired mutations for a target antibody modality. However, it is not yet clear if GSSPs provide a prospective mechanism for predicting likely antibody responses to specific immunogens. To address this question, a Markov Chain Monte Carlo simulation system is under development to simulate affinity maturation using GSSPs and using a structural bioinformatics module to evaluate the structural and functional effects of the introduced mutations. By simulating the maturation processes of hundreds to thousands of antibody clones, we expect to identify dominant maturation pathways for antigen-specific antibodies and to predict the likelihood of reproducing SHM patterns of interest. In the future, we will also build GSSPs for genes of animal models. We will be able to evaluate whether similar SHM patterns observed in human antibodies can be elicited in antibodies originated from homolog genes in animal models. Such analysis will be helpful for preclinical vaccine trials. Thus, GSSPs may thereby provide an important tool for rational vaccine design.

## Materials and Methods

### Donor Consent Information

Anonymized human PBMCs from normal, healthy donors were obtained through the NIH Clinical Center Department of Transfusion Medicine apheresis program by automated leukapheresis. Signed informed consent from the donors was obtained in accordance with the Declaration of Helsinki, and the study was approved by the National Institute of Allergy and Infectious Diseases (NIAID) Institutional Review Board. PBMCs were prepared by density gradient separation using Ficoll Paque Plus (GE HealthCare Life BioSciences, AB). Cells were then frozen in heat-inactivated fetal calf serum:DMSO (90:10) and stored at −185°C until needed.

### Next-Generation Sequencing

Immunoglobulin genes were amplified from PBMC samples from three HIV- and hepatitis C-negative individuals as previously described in Ref. (25, 42–44). Briefly, human PBMCs (6 × 10^7^) were previously obtained from three HIV-1 and hepatitis C-negative individuals (LP32647, LP08248 and LP23810), and the PBMCs were pelleted at 1,200 rpm for 8 min. mRNA was then extracted and eluted in 50 µl elution buffer using μMACS mRNA isolation kit (Miltenyi Biotec) according to the manufacturer’s instructions. The mRNA was then aliquoted for cDNA synthesis, and a 5′RACE approach was used to amplify IgG genes from one aliquot from each donor. The datasets were published and deposited in the NCBI Short Reads Archive (SRA ID: SRP067168) (25).

In this study, we prepared additional IgG libraries from aliquots of the same mRNA using 5′ RACE, as well as an IgG library using 5′ multiplex primers. In addition, we generated libraries for IgM, IgK, and IgL Ig genes using both 5′RACE or 5′multiplex methods. 5′RACE was conducted based on previously described methods (42). Briefly, to synthesize cDNA, 10 µl mRNA was mixed with 1 µl 5′CDS Oligo dT primers (12 µM) and incubated at 70°C for 1 min and then −20°C for 1 min. Then, 1 µl SMARTER Oligo Primer (12 µM) (Clontech), 4 µl 5× RT buffer, 1 µl DTT 20 (20 mM), 1 µl dNTP (10 mM), 1 µl RNAse out, and 1 µl SuperScript II reverse transcriptase (Invitrogen) were added to the reaction. After 2 h of incubation at 42°C, the cDNA products were purified using Nucleospin II kit (Macherey-Nagel) and eluted in 50 µl water. For Ig gene recovery, 10 µl cDNA was used for each PCR reaction. The first PCR amplification was performed with a common 5′ primer II A (Clontech) and Ig constant region-specific 3′ primer (IgG: 5′GGGGAAGACCGATGGGCCCTTGGTGG3′; IgM: 5′GAGGGGGAAAAGGGTTGGGGCGG3′, IgK: 5′GGAAGATGAAGACAGATGGTGCAGCCACAG3′, IgL: 5′CCTTGTTGGCTTGAAGCTCCTCAGAGGAGG3′) using KAPA HIFI qPCR kit (Kapa Biosystems). The PCR products were purified with 2% Size Select Clonewell E-gel (Invitrogen) and Agencourt AMPure XP beads (Beckman Coulter). The second PCR amplification was performed with primers with 454 sequencing adapters (454-RACE-F: 5′CCATCTCATCCCTGCGTGTCTCCGACTCAGAAGCAGTGGTATCAACGCAGAGT3′; 454-IgG-R: 5′CCTATCCCCTGTGTGCCTTGGCAGTCTCAGGGGGAAGACCGATGGGCCCTTGGTGG3′; 454-IgM-R: 5′CCTATCCCCTGTGTGCCTTGGCAGTCTCAGGAGGGGGAAAAGGGTTGGGGCGG3′; 454-IgK-R: 5′CCTATCCCCTGTGTGCCTTGGCAGTCTCAGGGAAGATGAAGACAGATGGTGCAGCCACAG3′; 454-IgL-R: 5′CCTATCCCCTGTGTGCCTTGGCAGTCTCAGCCTTGTTGGCTTGAAGCTCCTCAGAGGAGG3′). The PCR products were again purified with 2% Size Select Clonewell E-gel and Agencourt AMPure XP beads. For 5′ multiplex method, 10 µl mRNA was used for cDNA synthesis as described previously (43). cDNA was eluted in 50 µl water, and 10 µl cDNA was used for each PCR reaction. As described previously (43), IgG and IgM genes were amplified using mixed primers for all VH families, while IgK and IgL genes were amplified using mixed kappa and lambda primers, respectively. PCR products were purified with 2% Size Select Clonewell E-gel and Agencourt AMPure XP beads.

454 pyrosequencing for all libraries was performed as described previously (45), the datasets were deposited to NCBI SRA database (Bioprojects: PRJNA336331). The next-generation sequencing data for the antibody repertoires of the HIV infected donors NIH45, CAP256, and Z258 were published in Ref. (44) (SRA ID: SRP052625) (43, 46), (SRA ID: SRP034555 and SRP017087), and (Huang et al. Immunity 2016, Accepted) (SRA ID: SRR4417615-SRR4417632), respectively. The non-productive sequences derived from IGHV3-23 were retrieved from the European Nucleotide Archive (accession numbers AM076988-AM083316) (32).

### Quality Control and Generation of Non-Redundant Datasets

In order to ensure that our calculations were not affected by sequencing error from the 454 platform, we used a stringent quality control protocol as described in the following sections. We used SONAR^2^ to assign germline V and J genes for each transcript (27). Sequences for which assignments could not be made were removed. In addition, transcripts contained stop codons or out-of-frame junctions were also removed, as these are likely to be the result of sequencing error. Because members of an expanded clonal lineage are likely to share substitutions which did not arise independently, we included only one transcript from each lineage. Lineages were found using SONAR and defined as having the same V and J gene assignments and CDR3 sequences of the same length and at least 90% nucleotide identity. Lineages containing only a single transcript were discarded, as it is impossible to verify the quality of these sequences. For all lineages containing multiple sequences, the transcript closest to the consensus sequence of the lineage was chosen as the representative, which helps minimize the effects of sequencing error (44). In addition, because the primary mode of 454 error is indel, the remaining transcripts were each individually aligned to the assigned germline V gene with CLUSTALW to check for frameshift error. Any sequences with non-codon-length indels were discarded. Finally, each transcript was translated and compared to the amino acid sequence of the assigned germline V gene, and any transcripts with no amino acid changes were discarded, as they do not contribute any information to the GSSP. Paired-end reads obtained from donor Z258 using the Illumina MiSeq platform were merged into a full-length amplicons using USEARCH (47), discarding sequences containing > 25 mismatches in the overlap region. Merged reads with two or more expected errors (based on PHRED scores) were also discarded, and the remaining reads were processed in the fashion described earlier. Overall, we processed nearly 38 million NGS reads across 54 samples from six donors, which resulted in 14.6 million high-quality reads and over 200,000 lineages which we used to construct GSSPs (Table S1).

### Effects of Selecting a Representative Sequence from Each Lineage

Donor LP08248 had 284 VH3-23-derived lineages with at least 10 member sequences. We conducted 100 trials in which a random sequence was chosen as the representative of each lineage, and a mutability profile was constructed as described in the following sections. For each of these profiles, we calculated a set of rarity scores. We then calculated the Pearson’s correlation coefficient between the rarity scores of each pair of profiles; the distribution of these coefficients is shown in Figure S1B.

### Single Lineage Rarity Scores

We retrieved 348 VH3-30-derived sequences from the CAP256-VRC26 broadly HIV-1-neutralizing antibody lineage (46), including 33 experimentally isolated monoclonal antibodies. We constructed a GSSP from these sequences and compared the rarity scores derived from this profile to rarity scores derived from a VH3-30 profile constructed from the merged lineages of all three HIV^−^ donors (Figures S6C,D in Supplementary Material). For comparison, we also constructed a profile from 378 VH3-23 derived sequences from a single lineage found in donor LP08248 (Figures S6A,B in Supplementary Material).

### Identification of Potential Novel Germline Alleles

Identifying mutations requires an accurate knowledge of the germline sequence. Undocumented polymorphisms can appear as donor-specific, high-frequency mutations, skewing the resulting GSSPs and exaggerating differences between donors. We therefore used partis (21) to infer novel germline alleles from each non-redundant dataset. (Allele finding is considered a beta feature, available from https://github.com/psathyrella/partis. We used commit 610ae46 from June 18^th^, 2016.) Across all 6 donors, we found 33 unique novel heavy chain alleles, 23 novel kappa chain alleles, and 9 novel lambda chain alleles. Of these, 3, 5, and 4, respectively, were observed in two or more of these donors. The alleles have been deposited in the IgPdb^3^.

### Construction and Comparison of V Gene Substitution Profiles

For each non-redundant dataset, we used CLUSTALW2 to align translated transcripts from each V gene to all alleles of that gene, including novel alleles detected by partis. For each position, we calculated the substitution frequency and rarity as described in the text. For polymorphic positions, all possible polymorphisms were considered germline residues, without confirming the presence of each allele in each donor. Gaps and undefined amino acids were excluded from counting. GSSPs were only calculated if there were at least 100 sequences for a particular V gene in the given dataset. When building GSSPs, IMGT alleles IGHV4-4*07 and IGHV4-4*08 were considered as alleles of the IGHV4-59 gene, due to sequence similarity. Logo plots of GSSPs were shown using a customized version of WebLogo (48).

To compare two GSSPs, we calculated the Jensen–Shannon divergence between the distributions of mutations or substitutions in the two profiles at each position in the V gene. This is defined as

$$J(P_{A,i},P_{B,i})=H\left(\frac{P_{A,i}+P_{B,i}}{2}\right)-\frac{H(P_{A,i})+H(P_{B,i})}{2}$$

where *P_A,i_* and *P_B,i_* are vectors of length 20 describing the probability of observing each possible non-germline residue at position *i* for profiles *A* and *B*, respectively. *H* is the Shannon entropy, defined as

$$H(P_{i})=-\sum_{j=\{aa\}}P_{i}^{j}{\text{log}}_{2}P_{i}^{j}$$

where *j* can be any of the 20 amino acids. These position-wise Jensen–Shannon divergences were then averaged, with the divergence of each position weighted by the mean substitution frequency for that position in the two profiles being compared:

$$\langle J_{A,B}\rangle =\frac{\sum_{i}\frac{m_{A,i}+m_{B,i}}{2}J\left(P_{A,i},P_{B,i}\right)}{\sum_{i}\frac{m_{A,i}+m_{B,i}}{2}}$$

where *m_A,i_* and *m_B,i_* are the substitution frequency (observed frequency of mutation) at position *i* for profiles *A* and *B*, respectively. For comparisons of GSSPs from different V genes, only homologous positions were used. We generated a matrix of divergences among the datasets studied and used the cmdscale command in R to do multidimensional scaling and generate coordinates for plotting.

### Passenger Allele GSSP

The non-productive sequences were manually aligned to germline gene IGHV3-23 to remove indels in order to produce a meaningful GSSP. In cases where the boundaries of an indel were ambiguous, the boundary bases were replaced with Ns to avoid potential bias.

### Antibody Somatic Mutation Simulation

Starting from the nucleotide sequences of IGHV3-23*01, the S5F model was used to generate an artificial repertoire of 420 lineages, each with an independently simulated set of mutations. We produced 15 sequences each with 1–28 nucleotide changes; after accounting for the possibilities of silent mutations or multiple changes in a single codon, this produced a distribution of amino acid changes that roughly matched observed distributions of SHM in the functional repertoire (Figure S5C in Supplementary Material). For each sequence, we calculated the mutability of each nucleotide position under the S5F model and randomly selected a site for mutation based on those probabilities. The resulting substitution was then randomly chosen based on the substitution biases indicated for the target base by the S5F model. We excluded mutations that resulted in stop codons, but did not exclude re-mutation at a previously mutated site. For simulations with the AB model, positions to be mutated were selected using our own observations of mutation frequencies as calculated for the GSSP of IGHV3-23 and the substitution was then chosen under the AB model.

### Average Substitution Frequencies of the 20 Amino Acids

The GSSPs of 69 V genes (26 heavy chain, 20 kappa chain, and 23 lambda chain) were used to calculate the substitution frequencies. Because the substitution frequency at positions with low substitution frequency may be undersampled, we excluded positions being mutated in less than 5% of the V gene-specific clones. We also excluded positions containing amino acid changes among alleles. For each selected position of a profile, we normalized the substitution frequency to 1. Finally, all selected positions were grouped based on germline amino acid type. For each of the 20 amino acids, the substitution frequency to each of the other amino acids was averaged over all positions within a group.

### Statistical Test

The Mann–Whitney *U* test was used to evaluate the similarities of features between different groups in this study. Pearson’s correlations and statistical significance were calculated using cor.test in R.

## Ethics Statement

All work related to human subjects was performed in compliance with protocols approved by the NIH Institutional Review Board.

## Author Contributions

ZS, CS, PK, and LS designed research; ZS, CS, RK, JM, and LS performed research; ZS and CS analyzed data; ZS, CS, RK, JM, PK, and LS wrote the paper. All authors reviewed, commented on, and approved the manuscript.

## Consortia

The NISC Comparative Sequencing Program includes Betty Benjamin, Gerry Bouffard, Shelise Brooks, Holly Coleman, Mila Dekhtyar, Xiaobin Guan, Joel Han, Shi- ling Ho, Richelle Legaspi, Quino Maduro, Cathy Masiello, Jenny McDowell, Casandra Montemayor, James Mullikin, Morgan Park, Nancy Riebow, Jessica Rosarda, Karen Schandler, Brian Schmidt, Christina Sison, Ray Smith, Mal Stantripop, James Thomas, Pam Thomas, Meg Vemulapalli, and Alice Young.

## Conflict of Interest Statement

The authors declare that the research was conducted in the absence of any commercial or financial relationships that could be construed as a potential conflict of interest. The reviewer, PG, declared a shared affiliation, though no other collaboration, with the authors to the handling Editor, who ensured that the process nevertheless met the standards of a fair and objective review.

## Footnotes

^2^https://github.com/scharch/SONAR.

^3^http://cgi.cse.unsw.edu.au/~ihmmune/IgPdb/index.php.

## Supplementary Material

Figure S1 **Effects of sampling size on the robustness of GSSPs**. **(A)** For each of eight common VH genes, GSSPs were built using randomly sampled sets of 25, 50, 100, 200, or 300 clonal lineages. The Jensen–Shannon divergence was calculated between profiles from different donors but from the same gene and using the same number of lineages. Larger datasets resulted in lower Jensen–Shannon divergences, but the divergences converged when GSSPs were built using ~300 lineages, suggesting 300 lineages are required to build a robust GSSP. **(B)** To estimate the effects of the choice of representative sequence on GSSP construction, we randomly selected one representative sequence per lineage and built 100 repertoires. The rarity scores of mutations of randomly resampled repertoires showed an average correlation coefficient of ~0.98, suggesting that the rarity of mutations is robust to choice of representative sequences.

Figure S2 **Comparisons of the GSSPs of IGHV1-2 and IGHV1-69 and the distributions of SHM of IGHV1-2, IGHV1-69, and IGKV3-20 among three healthy donors**. **(A)** The distributions of amino acid SHM levels are similar among the three donors, as exemplified by IGHV1-2, IGHV1-69, and IGKV3-20. The Pearson correlation coefficients are listed, the correlations of which are all significant (*p* < 0.01). **(B)** The GSSP of IGHV1-2 constructed using lineages from the three healthy donors. **(C)** The GSSP of IGHV1-69 built using lineages from each of the three donors is noticeably different from those of IGHV1-2.

Figure S3 **GSSPs of codon AAG and CTG in various nucleotide contexts and correlation between substitution rarity and number of mutations within a codon**. **(A)** The GSSPs of codon AAG (encoding Lysine) are more similar at homologous positions (similar nucleotide context) than between non-homologous positions. **(B)** The GSSPs of codon CTG (encoding Leucine) are more similar at homologous positions than between non-homologous positions. Color scheme: green, amino acid replacement involves single nucleotide-mutation; yellow, amino acid replacement involves two nucleotide-mutations; red, amino acid replacement involves three nucleotide-mutations. **(C)** Amino acid mutations requiring more nucleotide substitutions within a codon tend to be rarer, partially explaining why many mutations are rare.

Figure S4 **Similarities of the GSSPs of V genes and over time**. For each of the three V gene types, **(A)** heavy chain, **(B)** kappa chain, and **(C)** labmda chain, the similarities measured by the Jensen–Shannon divergence and visualized using multidimensional scaling showed that the GSSPs of each V gene is similar among the three healthy donors, and V genes of the same family showed more similar GSSPs than between V families. We also observed that the GSSPs of a V gene are highly consistent over time for both **(D)** heavy chain and **(E)** kappa chain in donor NIH45.

Figure S5 **Similarities of substitution frequency and substitution bias among three healthy donors**. **(A)** The overall substitution frequency of all V genes are highly consistent between the three donors, indicating the substitution frequency of V gene is consistently modulated. **(B)** The overall rarity scores observed in all V genes are highly consistent among the three donors, suggesting the substitution bias is also consistently modulated. **(C)** Distributions of somatic hypermutation levels for VH3-23 lineages in the functional, passenger allele, and simulated repertoires shown in Figure 4.

Figure S6 **Similarity of rarity scores of 08248-00037 and CAP256-VRC26 lineages to the combined repertoire of the three healthy donors**. **(A)** The GSSP of the VH3-23-derived lineage 0037 from donor 08248 (bottom) compared to the overall GSSP of IGHV3-23 from the functional and passenger allele repertoires. **(B)** The correlation of rarity score between 08248-00037 and the overall GSSP of IGHV3-23 from the functional repertoire is non-significant. **(C,D)** The same plots for the VH3-30-derived broadly HIV-1-neutralizing lVRC26 lineage from donor CAP256 compared to the overall GSSP of IGHV3-30 from functional repertoires.

Figure S7 **Similarity of substitution frequencies and rarities at sites in the framework 3 region of IGHV3-23 compared by selection type detected by McCoy et al**. **(A)** Position-specific substitution rates calculated by McCoy et al were downloaded from doi:10.6084/m9.figshare.1399201 and rows containing “dNdS” estimates for the normalized “productive/out-of-frame” subset were extracted. As in Ref. (34), we considered only rows for which both “coverage” and “oof_coverage” were greater than 100. Sites with “hpdUpper” less than 1 were classified as being under negative selection and those with “hpdLower” greater than 1 were classified as being under positive selection. Sites under negative or positive selection showed a lowered correlation of substitution frequency between functional and non-productive repertoires, reflective of modulation by selection. **(B)** Sites classified as being under positive selection showed a significantly lowered correlation between rarity scores in functional and non-productive repertoires, suggesting selection modulates the substitution bias observed in GSSPs. Surprisingly, sites under negative selection showed increased correlation, possibly reflecting structural effects that SHM has evolved to avoid equally in functional and non-productive sequences.

Figure S8 **AB model compared to real data**. **(A)** The GSSP of the VH3-23 repertoire as simulated under the AB model (bottom) compared to the overall GSSP of IGHV3-23 from the functional and passenger allele repertoires. **(B)** The correlation of rarity scores between AB-simulated repertoires and the functional repertoire of donor 08248 (left) or non-productive passenger alleles (right).

Figure S9 **Correlations between substitution rarity and somatic hypermutation level**. **(A)** For each chain type, all transcripts in the three healthy donors were sorted into three groups based on SHM level, and the portions of rare mutations was estimated. The analysis showed that lineages containing higher SHM levels tend to have more rare mutation. **(B)** The substitution biases in the GSSP of low-SHM-lineages are similar to those of high SHM lineages (note different scales for *y*-axis). **(C)** Rarity for high- and low SHM lineages are highly correlated, though rarity calculated from low SHM sequences is uniformly higher, due to the lower overall substitution frequency. **(D)** The mutational space sampled by low-SHM-lineages is smaller but consistent with that of high-SHM-lineages.
