# Supplementary material for: Gene-Specific Substitution Profiles Describe the Types and Frequencies of Amino Acid Changes during Antibody Somatic Hypermutation
Source: Front Immunol. 2017 May 10;8:537. doi: 10.3389/fimmu.2017.00537 (PMC5424261; doi:10.3389/fimmu.2017.00537)
Supplement: Figure S1 — Effects of sampling size on the robustness of GSSPs. (A) For each of eight common VH genes, GSSPs were built using randomly sampled sets of 25, 50, 100, 200, or 300 clonal lineages. The Jensen–Shannon divergence was calculated between profiles from different donors but from the same gene and using the same number of lineages. Larger datasets resulted in lower Jensen–Shannon divergences, but the divergences converged when GSSPs were built using ~300 lineages, suggesting 300 lineages are required to build a robust GSSP. (B) To estimate the effects of the choice of representative sequence on GSSP construction, we randomly selected one representative sequence per lineage and built 100 repertoires. The rarity scores of mutations of randomly resampled repertoires showed an average correlation coefficient of ~0.98, suggesting that the rarity of mutations is robust to choice of representative sequences. [file Presentation_1.PDF]

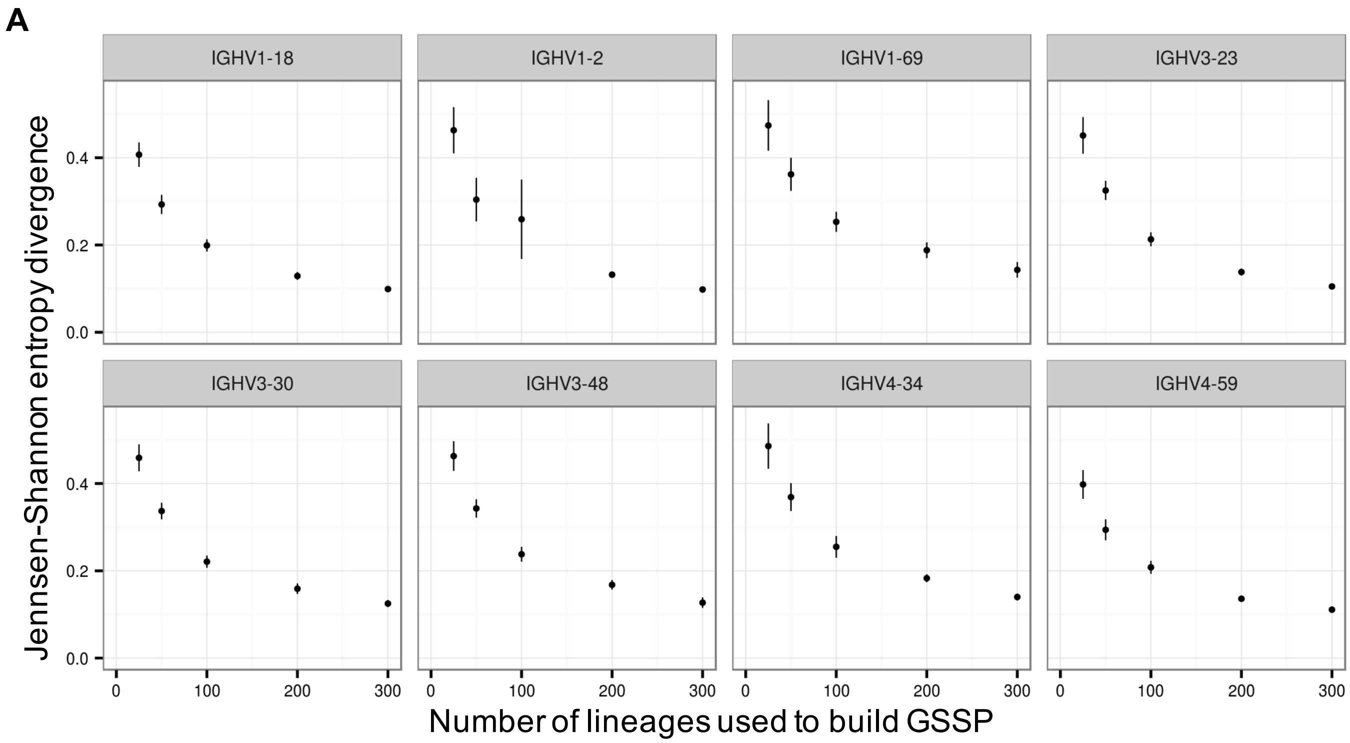

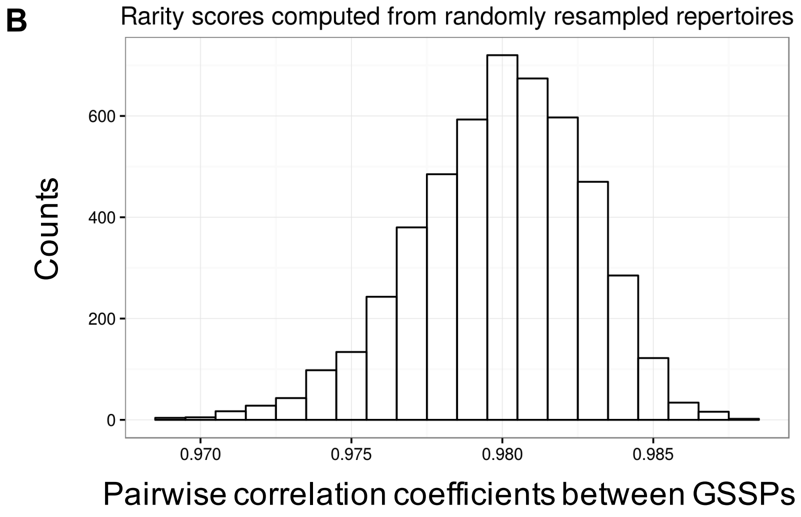

Figure S1. Effects of sampling size on the robustness of GSSPs. (A) For each of eight common VH genes, GSSPs were built using randomly sampled sets of 25, 50, 100, 200, or 300 clonal lineages. The Jensen-Shannon divergence was calculated between profiles from different donors but from the same gene and using the same number of lineages. Larger datasets resulted in lower Jensen-Shannon divergences, but the divergences converged when GSSPs were built using ~300 lineages, suggesting 300 lineages are required to build a robust GSSP. (B) To estimate the effects of the choice of representative sequence on GSSP construction, we randomly selected one representative sequence per lineage and built 100 repertoires. The rarity scores of mutations of randomly resampled repertoires showed an average correlation coefficient of ~0.98, suggesting the rarity of mutations is robust to choice of representative sequences.

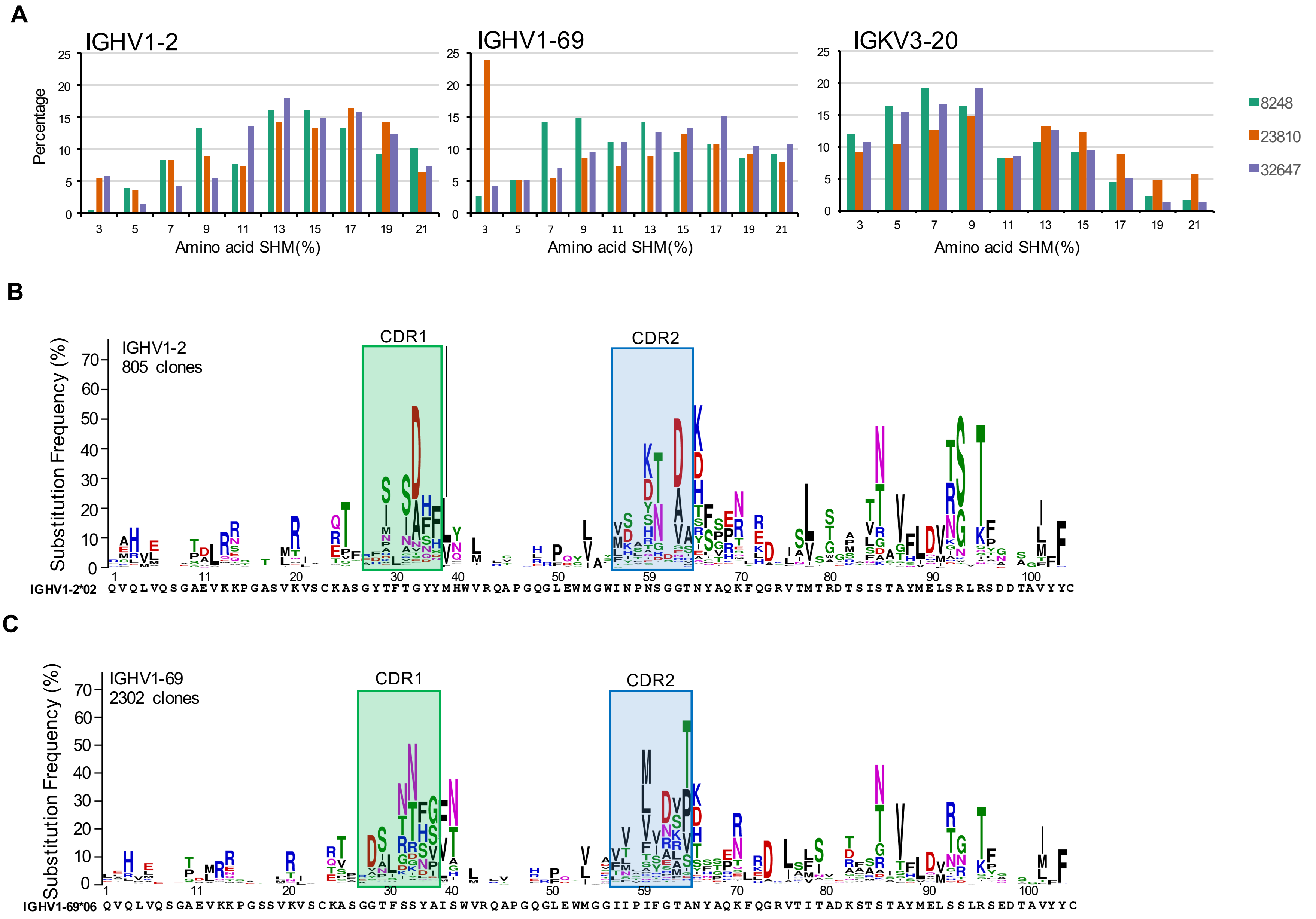

Figure S2. Comparisons of the GSSPs of IGHV1-2 and IGHV1-69 and the distributions of SHM of IGHV1-2, IGHV1-69, and IGKV3-20 among three healthy donors (A) The distributions of amino acid SHM levels are similar among the three donors, as exemplified by IGHV1-2, IGHV1-69, and IGKV3-20. The Pearson correlation coefficients are listed, the correlations of which are all significant (p<0.01). (B) The GSSP of IGHV1-2 constructed using lineages from the three healthy donors. (C) The GSSP of IGHV1-69 built using lineages from each of the three donors is noticeably different from those of IGHV1-2.

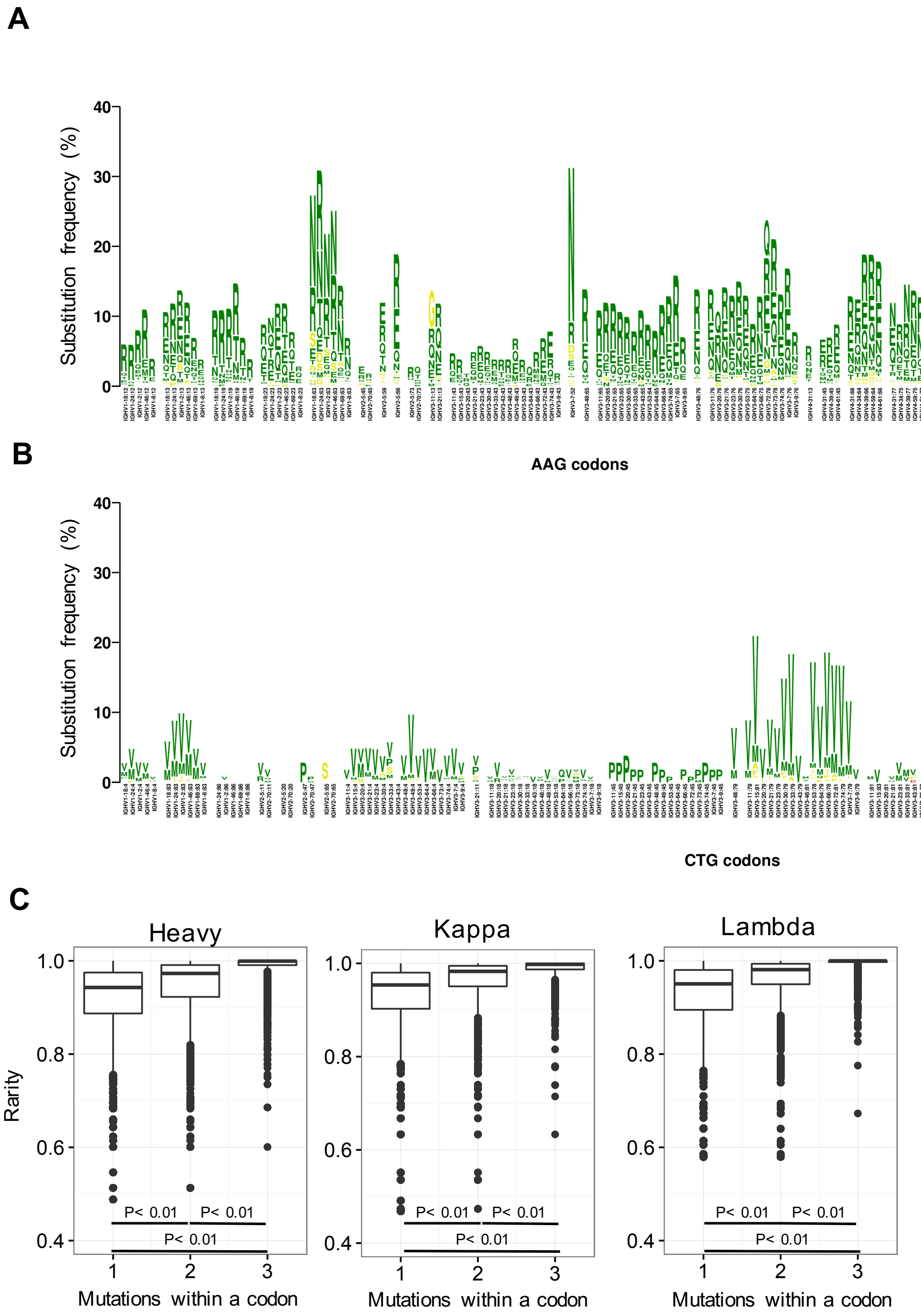

Figure S3. GSSPs of codon AAG and CTG in various nucleotide contexts and correlation between substitution rarity and number of mutations within a codon. (A) The GSSPs of codon AAG (encoding Lysine) are more similar at homologous positions (similar nucleotide context) than between non-homologous positions. (B) The GSSPs of codon CTG (encoding Leucine) are more similar at homologous positions than between non-homologous positions. Color scheme: green, amino acid replacement involves single nucleotide-mutation; yellow, amino acid replacement involves two nucleotide-mutations; red, amino acid replacement involves three nucleotide-mutations. (C) Amino acid mutations requiring more nucleotide substitutions within a codon tend to be rarer, partially explaining why many mutations are rare.

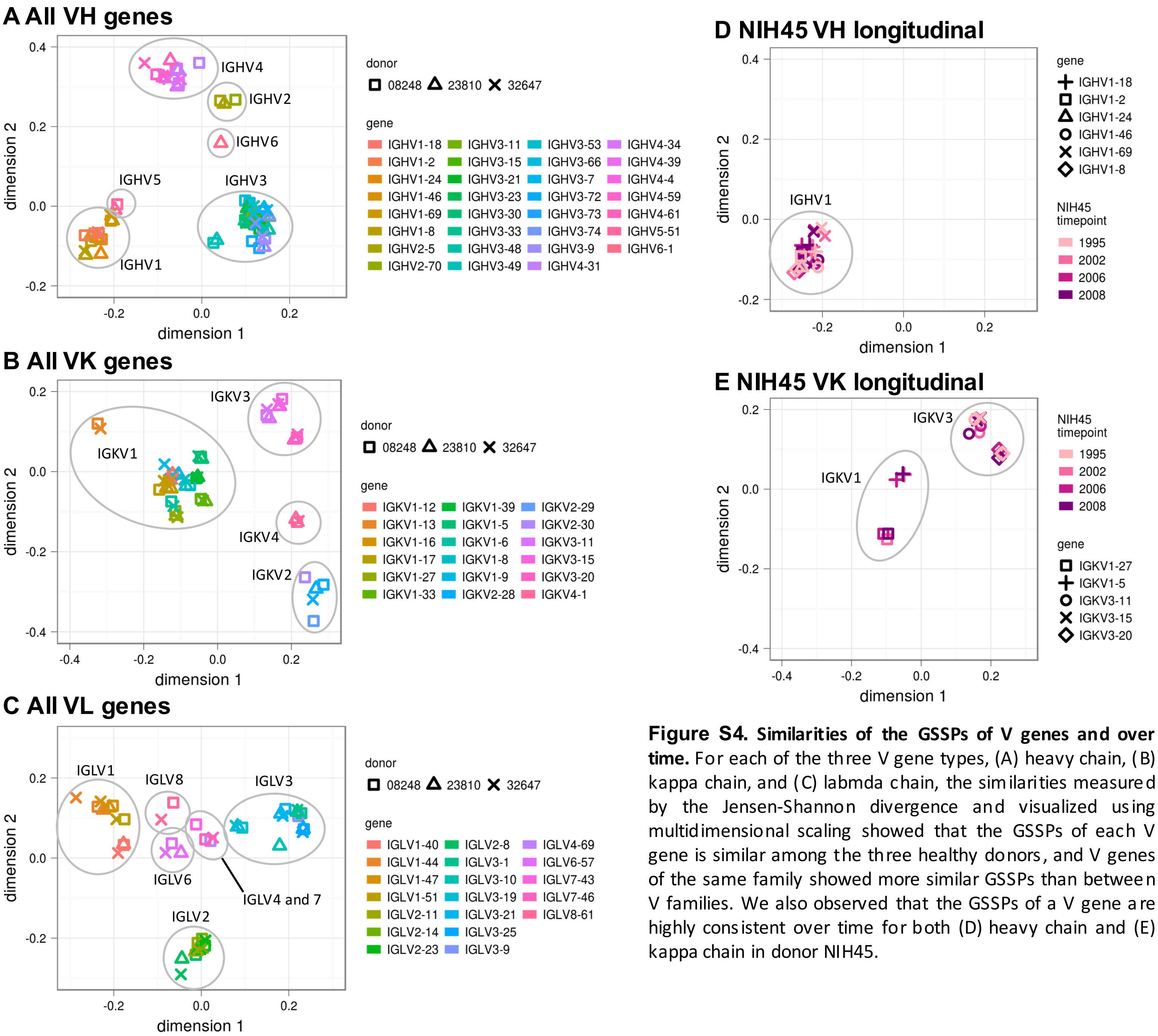

Figure S4. Similarities of the GSSPs of V genes and over time. For each of the three V gene types, (A) heavy chain, (B) kappa chain, and (C) labmda chain, the similarities measured by the Jensen-Shannon divergence and visualized using multidimensional scaling showed that the GSSPs of each V gene is similar among the three healthy donors, and V genes of the same family showed more similar GSSPs than between V families. We also observed that the GSSPs of a V gene are highly consistent over time for both (D) heavy chain and (E) kappa chain in donor NIH45.

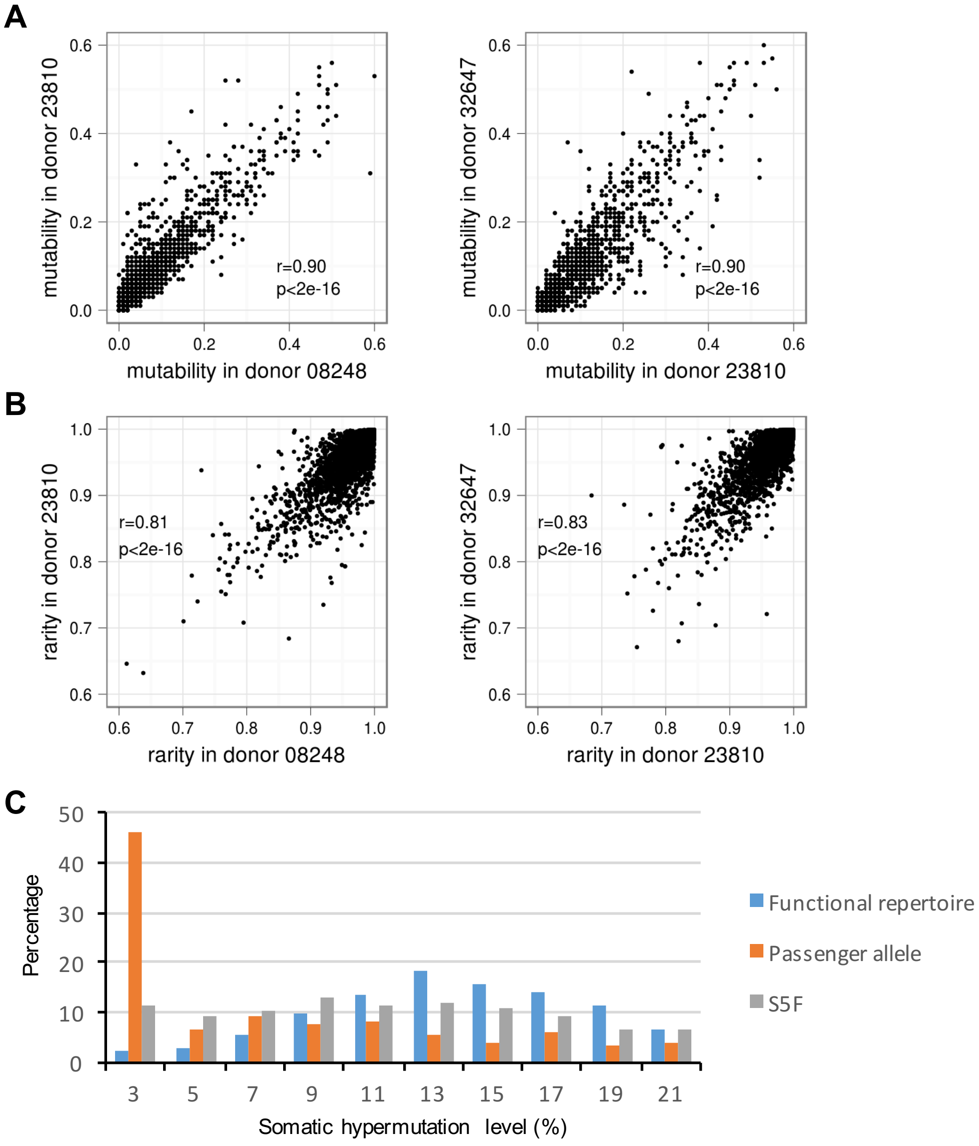

Figure S5. Similarities of substitution frequency and substitution bias among three healthy donors. (A) The overall substitution frequency of all V genes are highly consistent between the three donors, indicating the substitution frequency of V gene is consistently modulated. (B) The overall rarity scores observed in all V genes are highly consistent among the three donors, suggesting the substitution bias is also consistently modulated. (C) Distributions of somatic hypermutation levels for VH3-23 lineages in the functional, passenger allele, and simulated repertoires shown in Fig. 4.

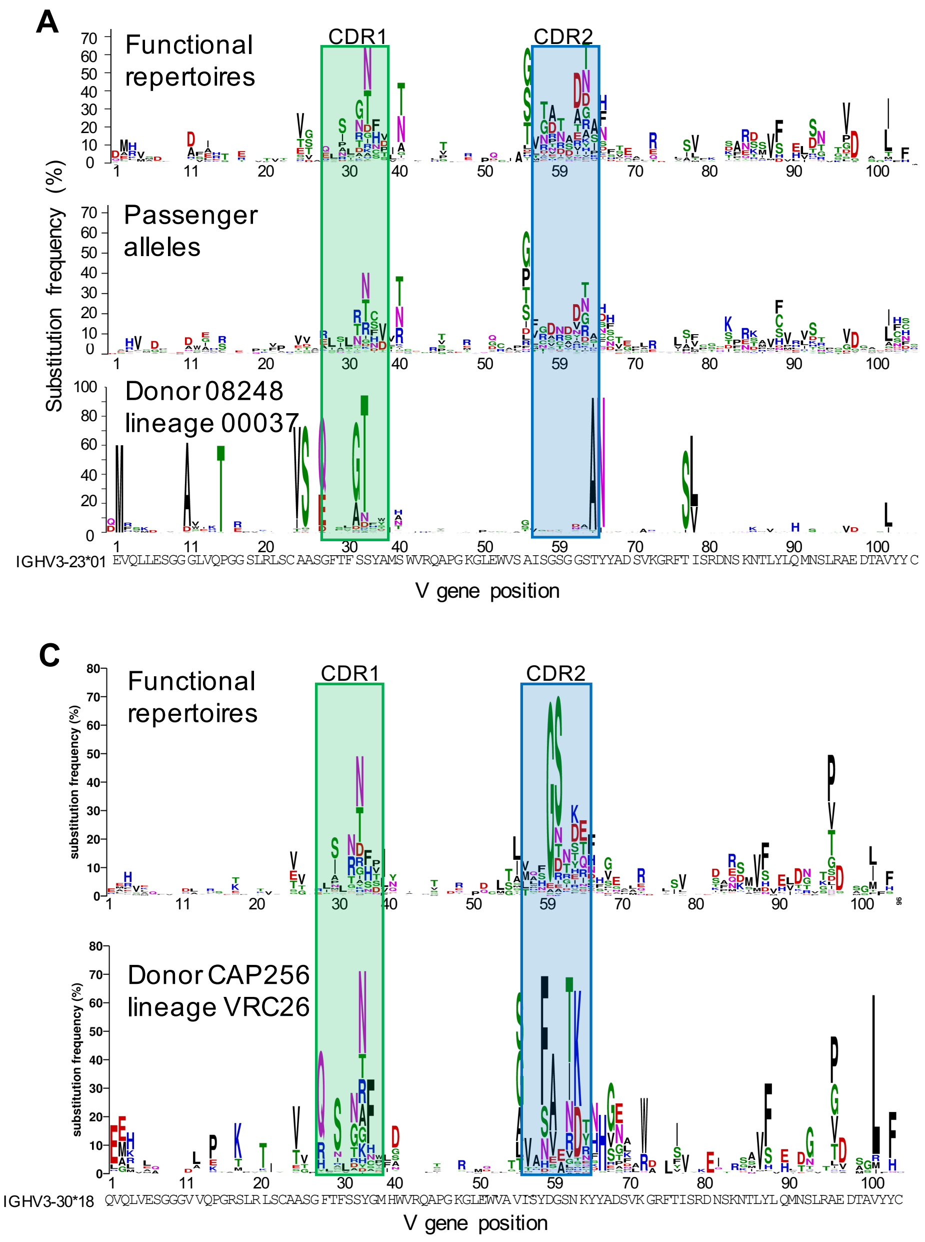

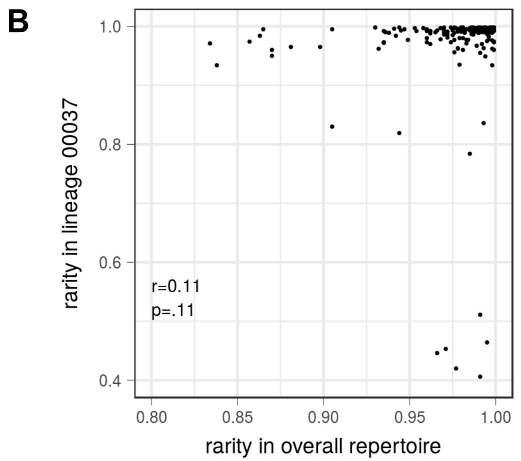

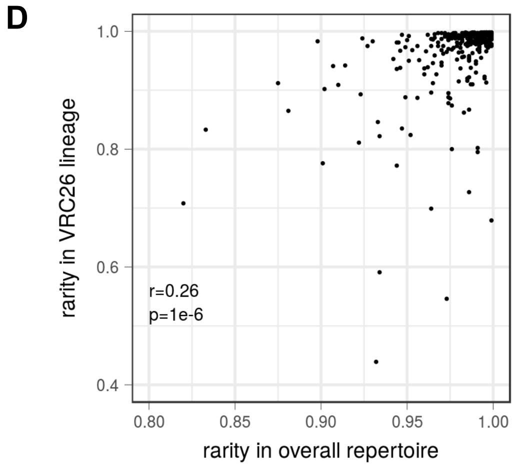

Figure S6. Similarity of rarity scores of 08248-00037 and CAP256-VRC26 lineages to the combined repertoire of the three healthy donors. (A) The GSSP of the VH3-23-derived lineage 0037 from donor 08248 (bottom) compared to the overall GSSP of IGHV3-23 from the functional and passenger allele repertoires. (B) The correlation of rarity score between 08248-00037 and the overall GSSP of IGHV3-23 from the functional repertoire is non-significant. (C) and (D) The same plots for the VH3-30-derived broadly HIV-1-neutralizing lVRC26 lineage from donor CAP256 compared to the overall GSSP of IGHV3-30 from functional repertoires.

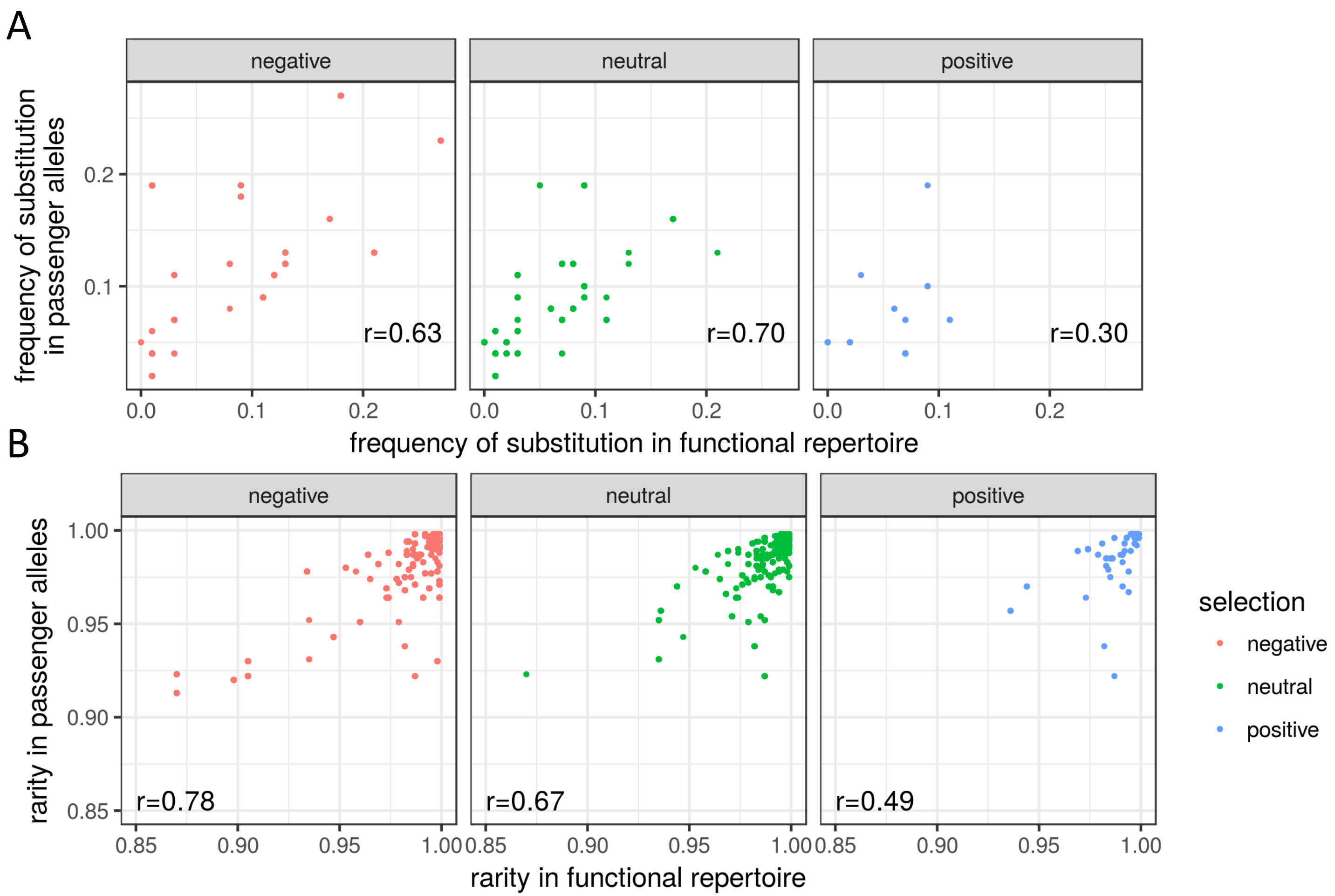

Figure S7. Similarity of substitution frequencies and rarities at sites in the framework 3 region of IGHV3-23 compared by selection type detected by McCoy et al. (A) Position-specific substitution rates calculated by McCoy et al were downloaded from doi:10.6084/m9.figshare.1399201 and rows containing 'dNdS' estimates for the normalized 'productive/out-of-frame' subset were extracted. As in McCoy study, we considered only rows for which both 'coverage' and 'oof_coverage' were greater than 100. Sites with 'hpdUpper' less than 1 were classified as being under negative selection and those with 'hpdLower' greater than 1 were classified as being under positive selection. Sites under negative or positive selection showed a lowered correlation of substitution frequency between functional and nonproductive repertoires, reflective of modulation by selection. (B) Sites classified as being under positive selection showed a significantly lowered correlation between rarity scores in functional and nonproductive repertoires, suggesting selection modulates the substitution bias observed in GSSPs. Surprisingly, sites under negative selection showed increased correlation, possibly reflecting structural effects that SHM has evolved to avoid equally in functional and nonproductive sequences.

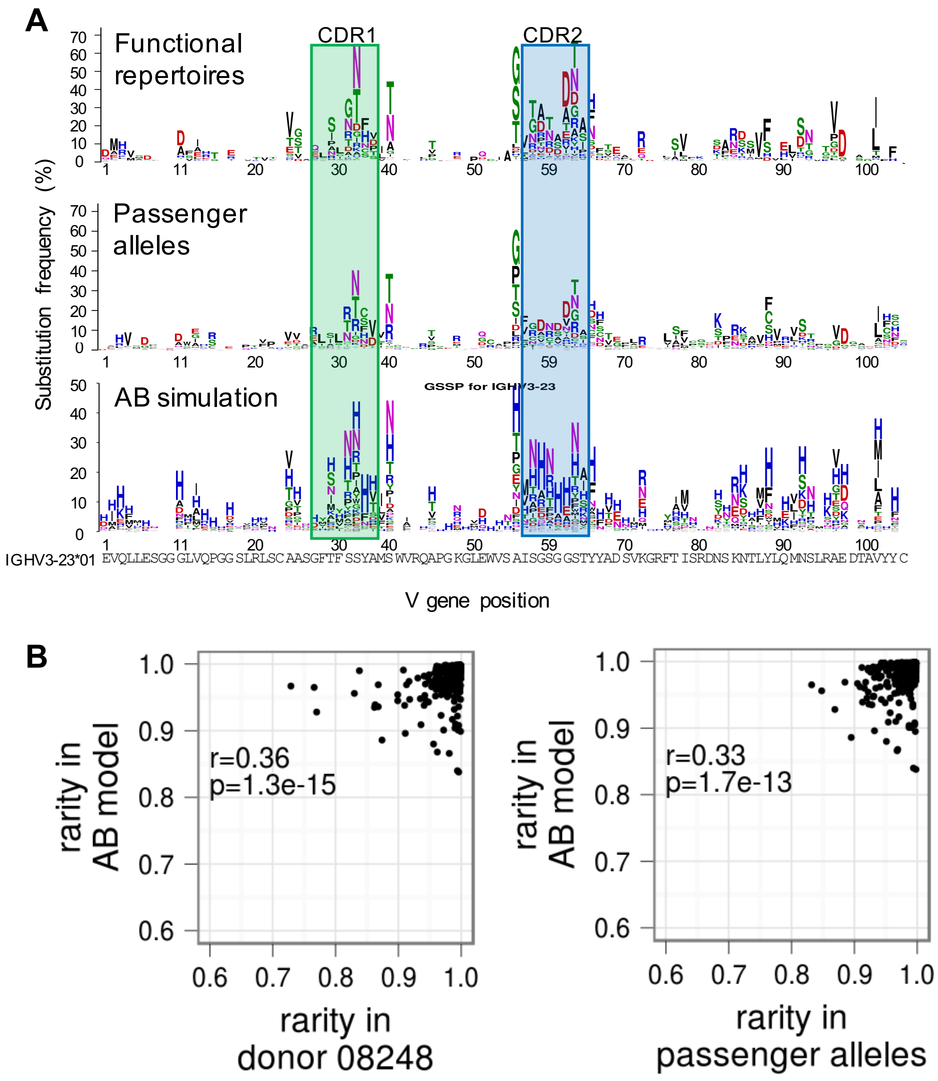

Figure S8. AB model compared to real data. (A) The GSSP of the VH3-23 repertoire as simulated under the AB model (bottom) compared to the overall GSSP of IGHV3-23 from the functional and passenger allele repertoires. (B) The correlation of rarity scores between AB-simulated repertoires and the functional repertoire of donor 08248 (left) or nonproductive passenger alleles (right).

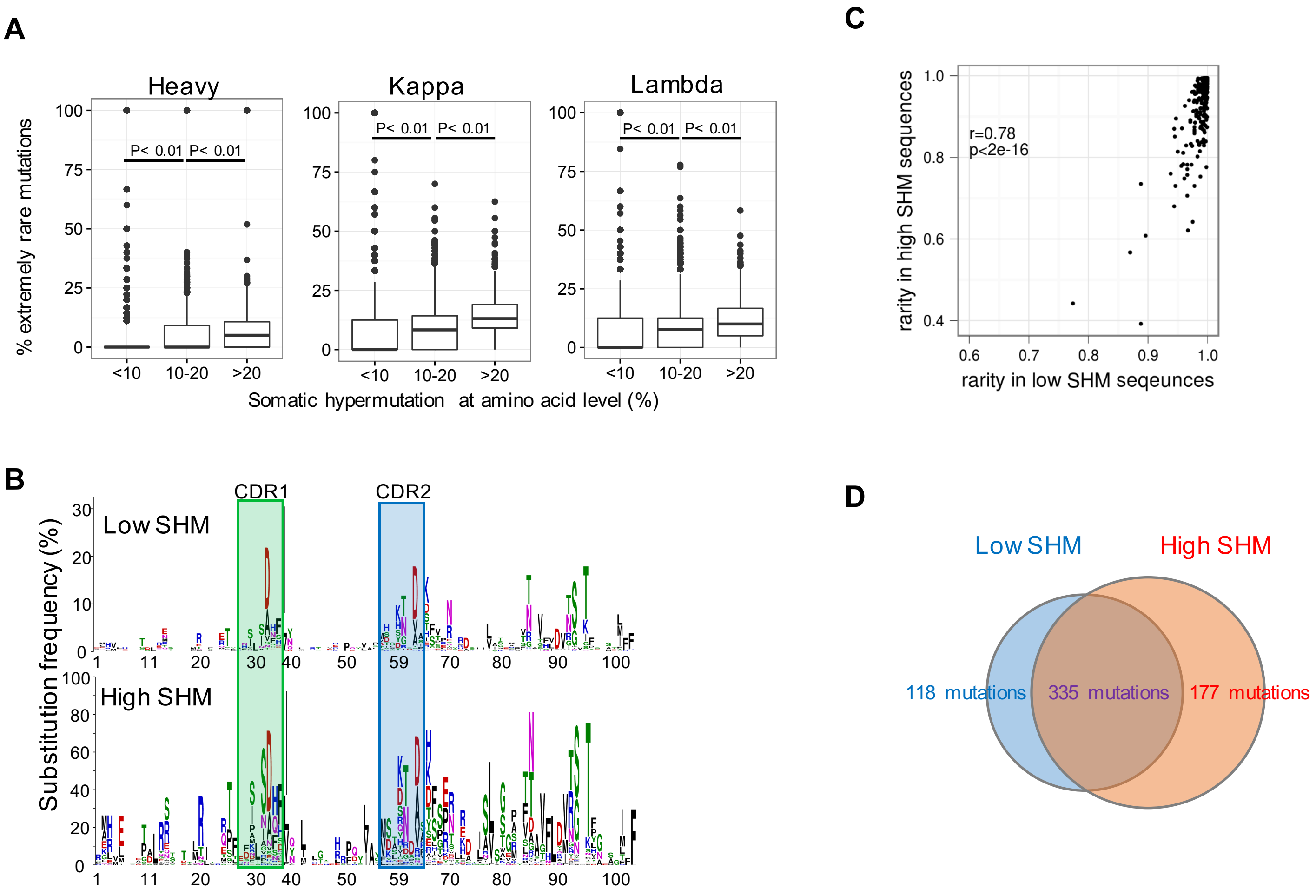

Figure S9. Correlations between substitution rarity and somatic hypermutation level. (A) For each chain type, all transcripts in the three healthy donors were sorted into three groups based on SHM level, and the portions of rare mutations was estimated. The analysis showed that lineages containing higher SHM levels tend to have more rare mutation. (B) The substitution biases in the GSSP of low-SHM-lineages are similar to those of high-SHM-lineages (note different scales for y-axis). (C) Rarity for high- and low-SHM-lineages are highly correlated, though rarity calculated from low SHM sequences is uniformly higher, due to the lower overall substitution frequency. (D) The mutational space sampled by low-SHM-lineages is smaller but consistent with that of high-SHM-lineages.
